# FUT8-Directed Core Fucosylation of N-glycans Is Regulated by the Glycan Structure and Protein Environment

**DOI:** 10.1021/acscatal.1c01698

**Published:** 2021-07-08

**Authors:** Ana García-García, Sonia Serna, Zhang Yang, Ignacio Delso, Víctor Taleb, Thomas Hicks, Raik Artschwager, Sergey Y. Vakhrushev, Henrik Clausen, Jesús Angulo, Francisco Corzana, Niels C. Reichardt, Ramon Hurtado-Guerrero

**Affiliations:** † Institute of Biocomputation and Physics of Complex Systems (BIFI), University of Zaragoza, Mariano Esquillor s/n, Campus Rio Ebro, Edificio I+D, Zaragoza 50018, Spain; ‡ Center for Cooperative Research in Biomaterials (CIC biomaGUNE), Basque Research and Technology Alliance (BRTA), Paseo Miramón 182, Donostia San Sebastián 20014, Spain; ∥ Copenhagen Center for Glycomics, Department of Cellular and Molecular Medicine, University of Copenhagen, Copenhagen DK-2200, Denmark; ⊥ School of Pharmacy, University of East Anglia, Norwich Research Park, Norwich NR4 7TJ, UK; # Departamento de Química Orgánica, Universidad de Sevilla, Sevilla 41012, Spain; ∇ Instituto de Investigaciones Químicas (CSIC-US), Avda. Américo Vespucio, 49, Seville 41092, Spain; ○ Departamento de Química, Universidad de La Rioja, Centro de Investigación en Síntesis Química, Logroño E-26006, Spain; ◆ CIBER-BBN, Paseo Miramón 182, San Sebastian 20014, Spain; ¶ Fundación ARAID, Zaragoza 50018, Spain

**Keywords:** FUT8, core fucosylation, N-glycosylation, STD NMR, enzyme kinetics, high-mannose N-glycans, complex N-glycans, paucimannose-type N-glycans

## Abstract

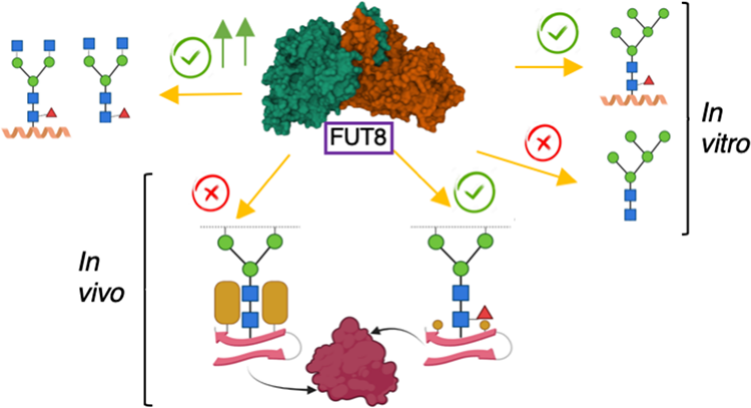

FUT8 is an essential α-1,6-fucosyltransferase that fucosylates the innermost GlcNAc of N-glycans, a process called core fucosylation. *In vitro*, FUT8 exhibits substrate preference for the biantennary complex N-glycan oligosaccharide (G0), but the role of the underlying protein/peptide to which N-glycans are attached remains unclear. Here, we explored the FUT8 enzyme with a series of N-glycan oligosaccharides, N-glycopeptides, and an Asn-linked oligosaccharide. We found that the underlying peptide plays a role in fucosylation of paucimannose (low mannose) and high-mannose N-glycans but not for complex-type N-glycans. Using saturation transfer difference (STD) NMR spectroscopy, we demonstrate that FUT8 recognizes all sugar units of the G0 N-glycan and most of the amino acid residues (Asn-X-Thr) that serve as a recognition sequon for the oligosaccharyltransferase (OST). The largest STD signals were observed in the presence of GDP, suggesting that prior FUT8 binding to GDP-β-l-fucose (GDP-Fuc) is required for an optimal recognition of N-glycans. We applied genetic engineering of glycosylation capacities in CHO cells to evaluate FUT8 core fucosylation of high-mannose and complex-type N-glycans in cells with a panel of well-characterized therapeutic N-glycoproteins. This confirmed that core fucosylation mainly occurs on complex-type N-glycans, although clearly only at selected glycosites. Eliminating the capacity for complex-type glycosylation in cells (KO *mgat1*) revealed that glycosites with complex-type N-glycans when converted to high mannose lost the core Fuc. Interestingly, however, for erythropoietin that is uncommon among the tested glycoproteins in efficiently acquiring tetra-antennary N-glycans, two out of three N-glycosites obtained Fuc on the high-mannose N-glycans. An examination of the N-glycosylation sites of several protein crystal structures indicates that core fucosylation is mostly affected by the accessibility and nature of the N-glycan and not by the nature of the underlying peptide sequence. These data have further elucidated the different FUT8 acceptor substrate specificities both *in vitro* and *in vivo* in cells, revealing different mechanisms for promoting core fucosylation.

## Introduction

N-glycan core fucosylation is post-translational modification (PTM) that takes place in most eukaryotes except for plants and fungi.^1^ This PTM is performed by a single fucosyltransferase (FUT) named FUT8.^1^ FUT8 is a Golgi resident-inverting α1-6-FUT that transfers a fucose residue from GDP-Fuc to the innermost GlcNAc moiety of N-glycans to form an α1-6-linkage.^1^ Core fucosylation is essential as revealed by early postnatal deaths with severe growth retardation and emphysema-like changes in the lung in mice with knockout of the *fut8* gene.^1^ Mutations in *FUT8* have also been found in humans, leading to a rare inherited metabolic disorder known as FUT8-CDG,^2^ which is characterized by a severe constellation of symptoms mimicking partly the symptoms found in the *fut8* knockout in mice. Core fucosylation is also strongly linked to cancer cell invasion and metastasis.^3^ FUT8 is up-regulated in a large number of cancer types,^3^ proposed to be due to at least three different mechanisms: (a) regulation of the expression of programmed cell death protein 1 (PD-1),^4^ (b) alteration of antibody-dependent cellular cytotoxicity (ADCC),^5^ and (c) regulation of transforming growth factor β1 receptor (TFG-β),^6^ epidermal growth factor (EGF) receptor,^7^ α3β1 integrin,^8^ and E-cadherin.^9^ Based on this, FUT8 is considered a promising drug target for the treatment of a large variety of cancer types.

*In vitro*, FUT8 prefers to fucosylate the biantennary complex N-glycan oligosaccharide (G0),^10^ requiring the presence of a terminal GlcNAc moiety on the α1-3 arm of the N-glycan with structural flexibility on the α1,6 arm.^11^ However, FUT8 can also fucosylate *in vitro* high-mannose N-glycopeptides that lack the terminal GlcNAc moiety on the α1-3 arm. In the latter case, a peptide/protein moiety attached to the innermost GlcNAc via an N-glycosidic linkage is essential since the lack of the peptide impairs core fucosylation.^12^ In addition, these findings are also supported by previous reports that demonstrate that some glycoproteins expressed from mammalian cells are core-fucosylated in high-mannose N-glycans.^13,14^ Presently, it has not been shown whether FUT8 recognizes the underlying peptide of the N-glycan oligosaccharide and whether this recognition favors glycosylation of complex N-glycopeptides versus complex N-glycan oligosaccharides. Furthermore, it is unknown to which extent high-mannose and paucimannose-type N-glycans are fucosylated *in vivo* or why apparently certain optimal *in vitro* N-glycan (e.g., biantennary complex N-glycan) substrates are not core-fucosylated *in vivo* in the context of a native protein.

**Figure 1:**
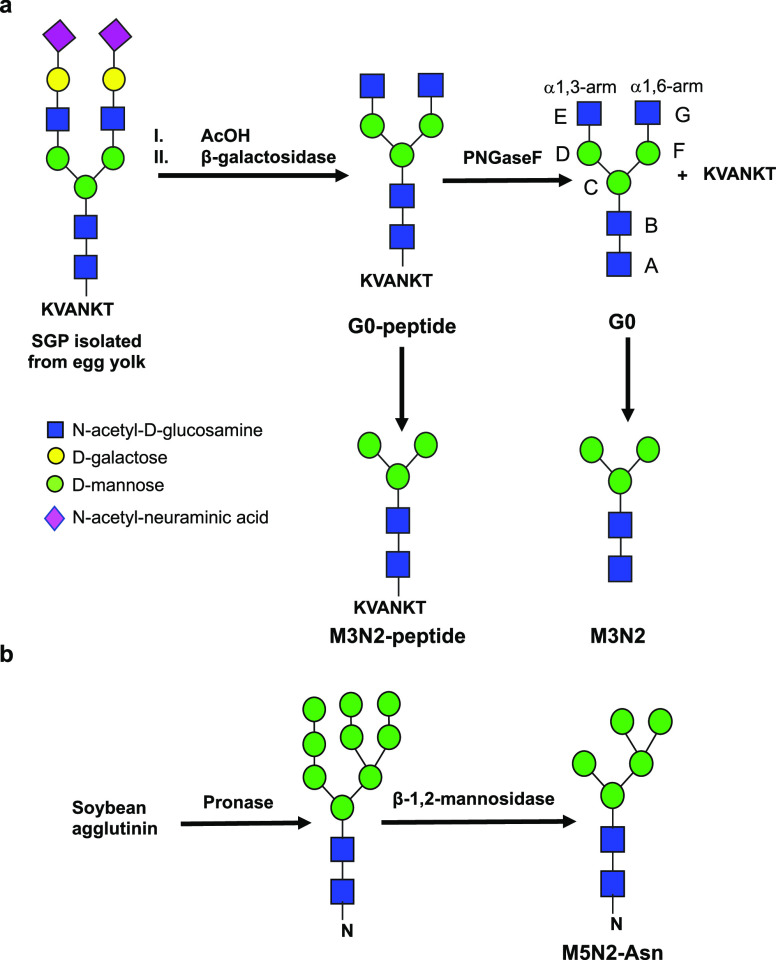
(a) Preparation of G0, the G0-peptide, M3N2, and the M3N2-peptide derived from the N-linked sialylglycopeptide isolated from egg yolk. (b) Preparation of M5N2-Asn from soy bean agglutinin. The nomenclature for G0 is also depicted.

FUT8 is a multidomain enzyme composed of an N-terminal coiled-coil domain, a GT-B fold catalytic domain, and an SH3 domain.^15^ The molecular basis of the substrate preference together with the catalytic mechanism has been recently elucidated by solving the crystal structures of FUT8 complexed to GDP and different Asn-linked oligosaccharides and G0.^10,16,17^ In the presence of GDP, FUT8 undergoes large conformational changes in several loops that contribute to the formation of the donor substrate binding site, which in turn brings the catalytic base Glu373 into the active site, a step required for catalysis. All sugar units of G0 are recognized by FUT8, particularly A**^G0^** and B**^G0^** by the catalytic domain and C**^G0^**, D**^G0^**, E**^G0^**, F**^G0^**, and G**^G0^** by an exosite (see Figure 1a for the nomenclature of the sugar units), which is formed by a loop connecting both the catalytic and SH3 domains, as well as the latter domain. This exosite and mainly the SH3 domain are proposed to fine-tune branch-specific acceptor affinity through both complementarity interactions and steric restrictions.^10,16−18^

To address the influence of peptides/proteins in core fucosylation of N-glycans, we report herein a multidisciplinary approach combining chemoenzymatic synthesis, enzyme kinetics, isothermal titration calorimetry (ITC), STD NMR spectroscopy, molecular dynamics (MD) simulations, and cell experiments applying genetic engineering to force high mannose-type N-glycosylation on recombinant glycoproteins. Our findings reveal that FUT8 only poorly glycosylates high-mannose or low-mannose N-glycopeptides such as paucimannose-type N-glycans, both *in vitro* and *in vivo*, and that an underlying peptide is required for glycosylation of these N-glycopeptides. Importantly, however, we do identify N-glycans on erythropoietin that are efficiently core-fucosylated as high-mannose glycans. Although we demonstrate that FUT8 recognizes all sugar units and also the OST sequon (Asn-X-Thr) of the G0-peptide, the kinetic and thermodynamic parameters of FUT8 on G0 and the G0-peptide are very similar. Furthermore, we show that FUT8 preferentially fucosylates complex N-glycans and that some N-glycosylation sites are not fucosylated *in vivo* because the protein substrate amino acids surrounding the innermost GlcNAc of the N-glycan likely hinder FUT8 binding.

## Results and Discussion

### Kinetics of FUT8 against Different N-glycan and Asn-Linked Oligosaccharides and N-glycopeptides

**Figure 2:**
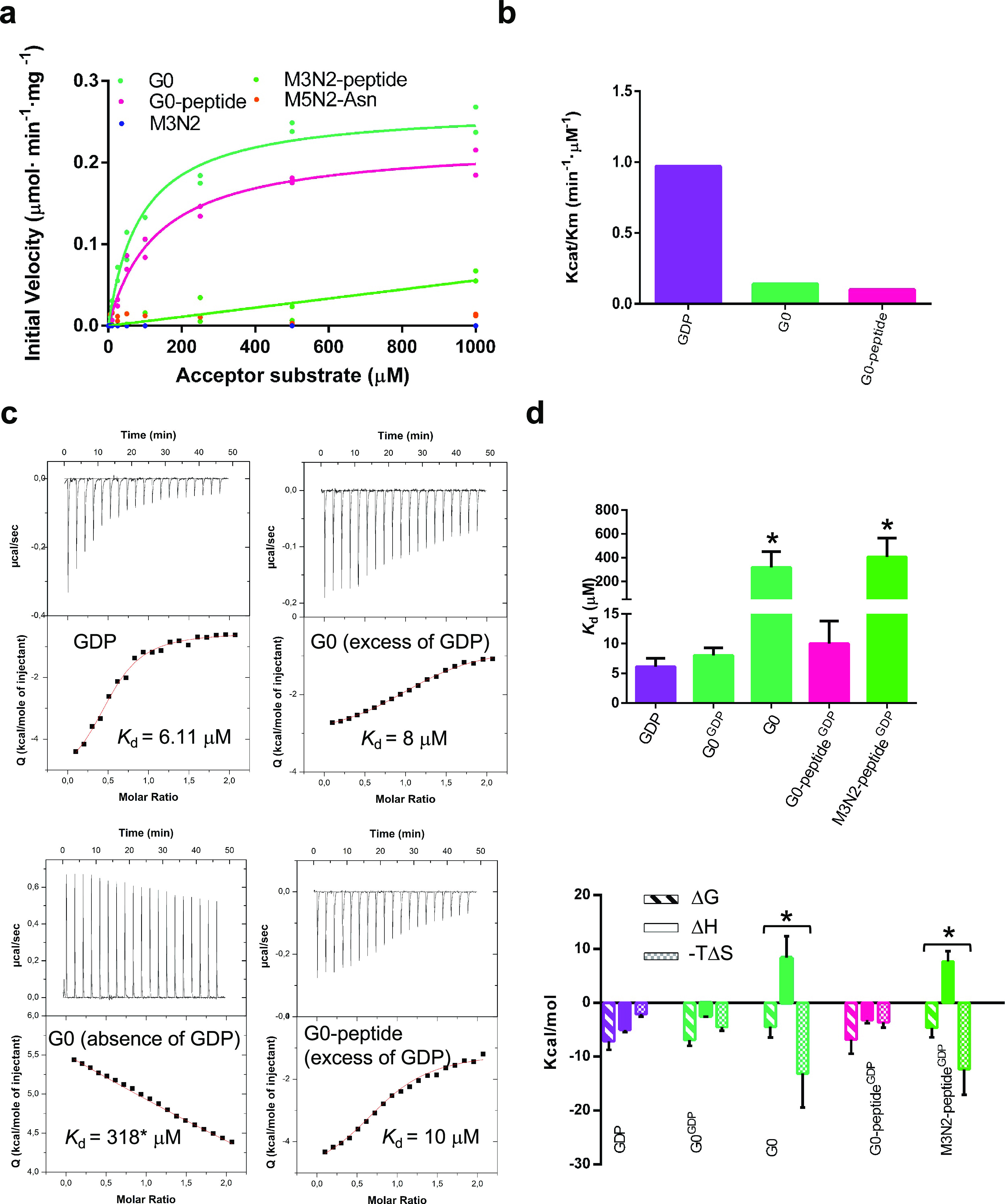
Enzyme kinetics and ITC experiments of FUT8 on diverse N-glycan and Asn-linked oligosaccharides and N-glycopeptides. (a) Glycosylation kinetics of FUT8 against the different acceptor substrates. (b) Plot comparing the catalytic efficiency (*k*_cat_/*K*_m_) of FUT8 against GDP, G0, and the G0-peptide. Additional kinetic data are given in Table 1. Note that kinetic parameters could not be obtained for the M3N2-peptide and M5N2-Asn. (c) ITC data for the binding of some of the ligands to FUT8. Top: raw thermogram (thermal power versus time). Bottom: binding isotherm (normalized heats versus molar ratio). (d) (Upper panel) Graph depicting the *K*_d_’s of the different enzyme forms. (Lower panel) Thermodynamic dissection of the interaction of the different enzyme forms with the different ligands. The binding Gibbs energy (Δ*G*), enthalpy (Δ*H*), and entropy (−*T*Δ*S*) are in kcal/mol. Any negative value represents a favorable contribution to the binding, whereas a positive value represents an unfavorable contribution. Asterisks (*) denote estimated values from the fitting.

To evaluate the role of the underlying peptide in core fucosylation of N-glycans, we synthetized a series of N-glycan and Asn-linked oligosaccharides and N-glycopeptides (see Figure 1 and Methods). From hen egg yolk, we isolated the N-linked sialylglycopeptide (SGP),^19^ with the peptide sequence “KVANKT”. This glycopeptide was further modified by acetic acid treatment to remove terminal sialic acids and galactose residues by the action of β-galactosidase to produce the G0-peptide. Further treatment of this glycopeptide with PNGaseF and β-*N*-acetylglucosaminidase produces G0, the Man_3_GlcNAc_2_ N-glycan oligosaccharide, and the Man_3_GlcNAc_2_ N-glycopeptide (Figure 1a). The Asn-linked oligosaccharide, Man_5_GlcNAc_2_-Asn, was obtained by the subsequent treatment of soybean agglutinin with Pronase and β-1,2-mannosidase (Figure 1b). Hereafter, and for simplicity, the latter three compounds will be named as M3N2, the M3N2-peptide, and M5N2-Asn, respectively. The identity of the compounds was verified by NMR and MALDI-TOF analyses (see the Supplementary Methods section and Figures S1–S4). We qualitatively determined the activity of FUT8 over the structures prepared by MALDI-TOF MS, showing that the G0-peptide was better core-fucosylated than the M3N2-peptide and M3N2, which were very poorly glycosylated under long incubation times (Figures S1–S3). Note that we previously demonstrated by MALDI-TOF MS that G0 was also a good substrate for FUT8.^16^ We then determined the FUT8 kinetic parameters of these compounds, allowing the determination of *k*_cat_, *K*_m_, and *k*_cat_/*K*_m_ for G0 and the G0-peptide (Figure 2a,b and Figure S5). Note that the kinetic parameters for GDP-Fuc were determined in the presence of a saturated concentration of G0. The *K*_m_’s for GDP-Fuc, G0, and the G0-peptide were 14.56 ± 3.4, 113.1 ± 15.43, and 133.1 ± 19.99, respectively, and the *k*_cat_ was ∼15 min^–1^ (Figure 2a and Table 1), a value in agreement with a previously reported *k*_cat_ value for FUT8 (*k*_cat_ of 24.6 min^–1^).^20^ The transfer reaction was also ∼10-fold more catalytically efficient for GDP-Fuc than that of G0. On the contrary and as expected from our MALTI-TOF analysis, FUT8 was slower against the M3N2-peptide (∼3.6-fold worse initial velocity than that of G0 and the G0-peptide at a 1 mM acceptor substrate; Figure 2a and Table 1), very slow against M5N2-Asn (∼14-fold worse initial velocity than that of the G0/G0-peptide at a 1 mM acceptor substrate), and inactive against M3N2. The data could not be fitted for FUT8 against the M3N2-peptide and M5N2-Asn to get reliable kinetic parameters because FUT8 was not saturated at higher concentrations of the acceptor substrates. Collectively, these data are further supported by a previous study showing that the biantennary complex N-glycan, G0-Asn, is a better substrate than M3N2-Asn and M5N2-Asn.^10^ In addition, a recent report also supports our findings that the kinetic parameters are very similar to those of G0 and the G0-peptide, implying that the peptide does not influence the kinetic parameters of the G0-peptide versus G0.^21^

**Table 1:** Kinetic Parameters for the FUT8 Glycosylation of the Different N-glycan and Asn-Linked Oligosaccharides and N-glycopeptides Used in This Study Using FUT8^d^

	*K*_m_ (μM)	*V*_max_ (nmol·min^–1^·mg^–1^)	*k*_cat_ (min^–1^)	*k*_cat_/*K*_m_ (min^–1^·μM^–1^)
GDP-Fuc^a^	14.56 ± 3.4	244.3 ± 13.1	14.17 ± 0.76	0.97
G0	113.1 ± 15.43	282.1 ± 11.72	15.62 ± 0.5	0.14
G0-peptide	133.1 ± 19.99	224.7 ± 10.68	13.03 ± 0.62	0.1
M3N2	b	b	b	b
M3N2-peptide	c	c	c	c
M5N2-Asn	c	c	c	c

a The *K*_m_ of GDP-Fuc was determined in the presence of a saturating concentration of G0.

b Not active.

c Kinetic parameters not determined (data could not be fitted to the nonlinear Michaelis–Menten equation because, under our conditions, FUT8 showed a linear increase in activity versus concentration of the N-glycans).

d Note that the first row defines the kinetic parameters for the donor substrate GDP-Fuc.

In short, while the presence of the peptide makes no difference in the kinetic parameters of FUT8 against G0 and the G0-peptide, it is clear that the peptide plays a critical role in the core fucosylation of the M3N2-peptide.

### Thermodynamic Parameters of FUT8 against the Different Acceptor Substrates

To determine the thermodynamic parameters of FUT8 against the different N-glycan and Asn-linked oligosaccharides and N-glycopeptides, we performed isothermal titration calorimetry (ITC) experiments. First, we determined the *K*_d_ of GDP for binding to FUT8 (*K*_d_ = 6.1 ± 1.4 μM; Table 2 and Figure 2c,d, upper panel). Then, we evaluated whether this enzyme requires prior GDP binding to G0. While, in the absence of GDP, FUT8 showed very poor binding to G0 (*K*_d_ = 318 ± 139 μM), this turned out to be the opposite in the presence of GDP (*K*_d_ = 8 ± 1.3 μM; binding to G0 was ∼40-fold better in the presence of GDP than in its absence; Figure 2c,d and Table 2). The poor binding of FUT8 to G0 in the absence of GDP is also supported by previous SPR data that rendered a *K*_d_ of 390 μM.^22^ These data provide compelling evidence that FUT8 likely follows an ordered bi–bi kinetic mechanism. In this mechanism, which is further supported by previous crystal structures of FUT8 complexes,^10,16,17^ the enzyme is in an inactive state in the apo form (open loops) and shifts to the active state (closed loops) in the presence of GDP-Fuc. This mechanism also implies an induced-fit mechanism by GDP-Fuc that has been recently proposed,^10^ where the sugar nucleotide would induce the closure of several loops, leading to an active state conformation. The induced-fit mechanism has become a general mechanism for glycosyltransferases (GTs) and has been also proposed for distant GTs such as GalNAc-T2, B4GALT1, and lactose synthase.^23−25^

**Table 2:** Thermodynamic Parameters for N-glycan and Asn-Linked Oligosaccharides and N-Glycopeptides Binding to FUT8^c^

	*K*_d_ (μM)	Δ*G* (kcal/mol)	Δ*H* (kcal/mol)	–*T*Δ*S* (kcal/mol)	*n*
GDP	6.1 ± 1.4	–7.09 ± 1.65	–5.02 ± 0.4	–2.07 ± 0.48	0.60
G0 (excess GDP)	8 ± 1.3	–6.89 ± 1.11	–2.42 ± 0.15	–4.47 ± 0.72	1.2
G0 (without GDP)	318 ± 134^b^	–4.75 ± 2	8.35 ± 4.05	–13.1 ± 6.35	1.6
G0-peptide (excess GDP)	10 ± 4	–6.82 ± 2.6	–3.2 ± 0.56	–3.62 ± 0.99	0.84
M3N2-peptide (excess GDP)	406 ± 159^b^	–4.6 ± 1.8	7.7 ± 1.9	–12.3 ± 4.8	1.6
M3N2 (excess GDP)	a	a	a	a	a
M5N2-Asn (excess GDP)	a	a	a	a	a

a Not measurable under our conditions. This might be due to the fact that the binding is very weak.

b Estimated values from the fitting.

c *K*_d_ is the dissociation constant (=1/K), and Δ*G*, Δ*H*, and −*T*Δ*S* are the thermodynamic parameters. Stoichiometry of binding in all cases was close to ∼1:1. Except for the first ITC experiment in which the *K*_d_ was determined for GDP in the presence of FUT8, the rest of the ITCs were performed with the N-glycans and N-glycopeptides in the absence or presence of GDP.

Having established that the presence of GDP leads to a better binding of FUT8 to G0, we determined the thermodynamic parameters for the G0-peptide, M3N2, the M3N2-peptide, and M5N2-Asn in the presence of an excess of GDP. As we found out for the highly similar kinetic parameters between G0 and the G0-peptide, the *K*_d_’s for G0 and the G0-peptide did not show significant differences, implying that the peptide mostly in the G0-peptide is not important for the overall binding. However, contrary to the G0 versus G0-peptide parameters, FUT8 was bound to the M3N2-peptide (*K*_d_ = 406 ± 159 μM) and not to M3N2 under our conditions (Table 2 and Figure S6). In fact, we could not determine a *K*_d_ for the M3N2 glycan (Table 2). This clearly suggests that the peptide mostly may play a key role in binding to the M3N2-peptide and is likely behind the reason why the M3N2-peptide is a substrate for FUT8 despite its poor affinity. Furthermore, we also could not observe a binding titration profile for M5N2-Asn, likely explaining why this was one of the worst substrates for FUT8.

A detailed analysis of the thermodynamic parameters showed that the binding of the different acceptor substrates to FUT8 was entropy-driven (−*T*Δ*S*), while the binding of GDP was favored by a gain in enthalpy (Δ*H*), with a reduced entropic component (Figure 2d, lower panel, and Table 2), implying distinct interaction behaviors between these molecules. The unique thermodynamic profile exhibited by the different acceptor substrates might be due to the release of a vast number of surface water molecules from the FUT8 surface upon acceptor binding, promoting favorable desolvation entropy. On the contrary, the significant reduction in GDP-Fuc donor substrate mobility upon binding to the enzyme and the large number of hydrogen bonds between GDP to FUT8 are largely the major factors explaining the reduction in the entropic component and the favorable enthalpy.

We conclude that although the peptide on the G0-peptide does not play a significant additional role in FUT8 turnover and binding, the presence of the peptide on the M3N2-peptide is key for its binding and catalysis. These differences could be attributed to G0 per se being already a good binder to FUT8, implying that the peptide might not contribute much to binding. However, M3N2 is a poor binder whose binding to FUT8 is significantly improved by the presence of the peptide, enhancing FUT8 binding and core fucosylation.

### Molecular Dynamics (MD) Simulations and NOESY-Based NMR against the M3N2-Peptide and M3N2

A recent report^21^ hypothesized that the presence of the peptide might drive the *anti*-ψ conformation for the core-chitobiose GlcNAc moieties of the N-glycan in the free state. This conformer, which is less stable relative to the typical *syn*-ψ conformation found in solution, was previously demonstrated to be required for FUT8 recognition and catalysis.^16^ To determine the veracity of that hypothesis, we ran molecular dynamics (MD) simulations as well as NOESY-based NMR experiments to calculate experimental internuclear average distances on M3N2 and the M3N2-peptide.

Simulations for both M3N2 and the M3N2-peptide were produced starting either from the *syn* or *anti* conformers of the chitobiose core. The *anti* conformers rapidly flipped to the more energetically stable *syn* conformation, being the preferred one along the four simulations (Figure S7). Furthermore, to get experimental evidence of the proposed conformation, internuclear average distances were determined from the initial slopes of transient NOE build curves (Figure S8). Several 2D-NOESY experiments with increasing mixing time were acquired for both M3N2 and the M3N2-peptide. Signal overlapping in the M3N2 spectra made impossible the integration of enough isolated signals to calculate any distance. However, in the case of the M3N2-peptide, it was possible to obtain build-up curves for several proton pairs, and initial growth slopes were calculated (Figure S9). Reference distances H1A-H5A and H1B-H5B were extracted from the MD simulation for greater accuracy (Figure S10), being 2.58 Å in both cases. Interglycosidic distances H1B-H4A and H1B-H5A were then determined using the isolated spin pair approximation, giving values of 2.60 and 3.70 Å, respectively, which are compatible only with a GlcNAc-β1,4-GlcNAc *syn* conformation (Figure S11), therefore demonstrating that the peptide does not induce the energetically unfavorable *anti* conformation.

### STD NMR Spectroscopy of FUT8 against the G0-Peptide, the M3N2-Peptide, M3N2, and the Naked Peptide

Having established that the peptide does not lead to the unfavorable *anti* conformation in the free state, we performed STD NMR experiments to shed light on the role of the peptide in core fucosylation. STD NMR allows mapping of the binding epitopes of ligands in complex with large molecules. Initially, we determined that the naked peptide itself does not interact with FUT8 either in the absence or presence of GDP, implying that the peptide is likely recognized by FUT8 only in the context of an N-glycopeptide (see Methods and Figure S12). Next, we performed STD NMR on FUT8 against M3N2 and the M3N2-peptide under the same conditions described above (Figures S13 and S14). Again, no STD NMR signals were observed likely due to the poor affinity of these ligands to FUT8. Since we could not obtain insights from the interaction of the peptide with FUT8 using the peptide alone or the M3N2-peptide, we decided to perform additional STD NMR experiments using the G0-peptide. The G0-peptide indeed clearly displayed STD signals (see below), and the STD effects for the G0-peptide were measured under increasing NMR saturation times, producing build-up curves that were used to calculate the build-up rates, which were normalized with the highest value of 100% assigned (Figure S15). This procedure was carried out in the absence and presence of GDP to determine whether prior GDP binding to FUT8 leads to changes in G0-peptide recognition.

**Figure 3:**
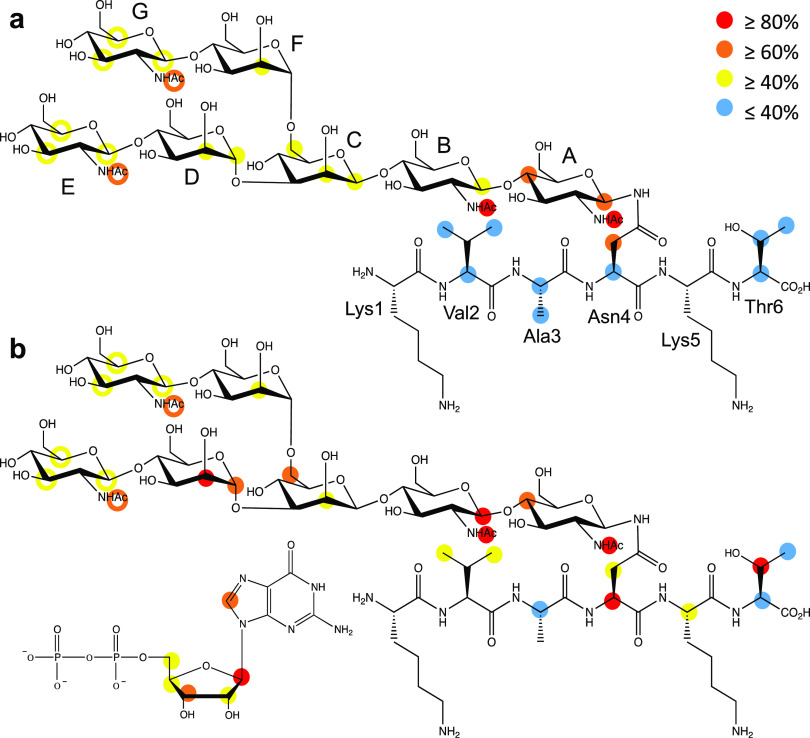
Binding epitope mapping of the G0-peptide with FUT8 by STD NMR in (a) the absence and (b) presence of GDP. Protein saturation was achieved by irradiation at −0.50 ppm. The colored circles represent the normalized STD-NMR intensity. Only STD responses are indicated for protons that could be accurately measured. Hollow circles indicate the sum of STD intensities of overlapping GlcNAc^E^ and GlcNAc^G^.

In STD NMR, the higher the STD effect, the closer the distance of the observed proton is to the protein surface (Methods, Figure 3, and Figure S16). As the STD signals arising from protons of the GlcNAc^E^ and GlcNAc^G^ moieties overlapped, the analysis is based on the average of the STD effects of both residues. In the absence of GDP (Figure 3a), a moderate STD effect is observed for every sugar ring, particularly H1 and H4 in GlcNAc^A^, which shows the highest saturation transfer. Remarkably, all the four acetyl groups showed high STD values, especially those on GlcNAc^A^ and GlcNAc^B^. The peptidic moiety showed low values, along the whole fragment (KVANKT), with only significant STD signals on Asn4, the glycosylated residue. This STD NMR binding epitope map qualitatively matches to that inferred from the previously described crystallographic structure of FUT8 complexed to G0 (PDB entry: 6TKV) where the whole heptasaccharide fits into a Y-shaped groove and GlcNAc^A^ is the most intimately recognized sugar by FUT8.^16^

When the STD NMR experiment is acquired in the presence of GDP (Figure 3b), a general increase in the STD effect is observed for all proton signals. This increase is particularly remarkable for sugar units A, B, C, and D. Although, from the crystal structure, it was suggested that FUT8 recognized GlcNAc^E^ better than GlcNAc^G^,^16^ we could not infer that from our STD NMR experiment because the STD signals for GlcNAc^E^ and GlcNAc^G^ overlapped in the spectra. The peptide protons also showed an increase in STD effects, especially for Asn4 and Thr6 and, to a lesser extent, Val2, pointing a closer interaction of the peptide with FUT8 in the presence of GDP. However, as shown before, FUT8 did not show differences in affinity between G0 and the G0-peptide, implying that the peptide did not contribute to the overall optimal binding of G0 toward FUT8 (Figure 3b). The STD effects on GDP showed a similar pattern to that found in the crystal structure of the FUT8-GDP-G0 complex.^16^

We then ran 0.5 μs molecular dynamics (MD) simulations on the G0-peptide and the M3N2-peptide bound to FUT8 in the presence of GDP, which showed that the peptide was relatively flexible in both complexes. This might explain the finding that after numerous crystallographic attempts to determine the structure of FUT8 complexed to GDP and the G0-peptide, we could not obtain the density for the peptide. The most populated hydrogen bond between the peptide moiety and the enzyme was established between the side chain of the glycosylated Asn4 and Gly217. In the G0-peptide, the side chain of Lys5 interacts with Glu373, and in the M3N2-peptide, a low-populated hydrogen bond engaging the side chain of Thr6 and Asp368 was found. Notably, this interaction could stabilize the flexible loop comprising Asp368 and Glu373, which is required for the catalysis and may explain why the peptide fragment in M3N2 enhances fucosylation (Figures S17 and S18).

In short, our STD NMR measurements suggest that the two parts of the N-glycopeptide can interact with FUT8. However, the peptide showed much lower STD values, indicating longer distances to the protein surface, implying that the peptide is more dynamic than the glycan as we found in our MD simulations. The presence of GDP in the complex does not dramatically change the binding epitope or the protein surface around the N-glycan. Yet, it enhances the interactions of FUT8 with most of the sugar units and the C-terminal residues of the peptide, further supporting the differences found in *K*_d_’s for G0 in the absence and presence of GDP. Furthermore, the finding that the peptide of the M3N2-peptide is clearly recognized by FUT8 provides a plausible explanation as to why the peptide of the M3N2-peptide might enhance its binding to FUT8 and in turn promote core fucosylation. Interestingly, FUT8 preferably recognizes the Asn-Lys-Thr peptide sequence, which matches the sequon found for OST, a multimeric complex that transfers a preassembled oligosaccharide to selected asparagine residues within the consensus sequence Asn-X-Ser/Thr.^26^ This also implies that these two very distant GTs with different structures and donor/acceptor substrates likely share the same sequon and that FUT8 also recognizes additional amino acids beyond the previously proposed result, suggesting that FUT8 might only recognize the Asn residue.^21^

### Core Fucosylation in Cells

**Figure 4:**
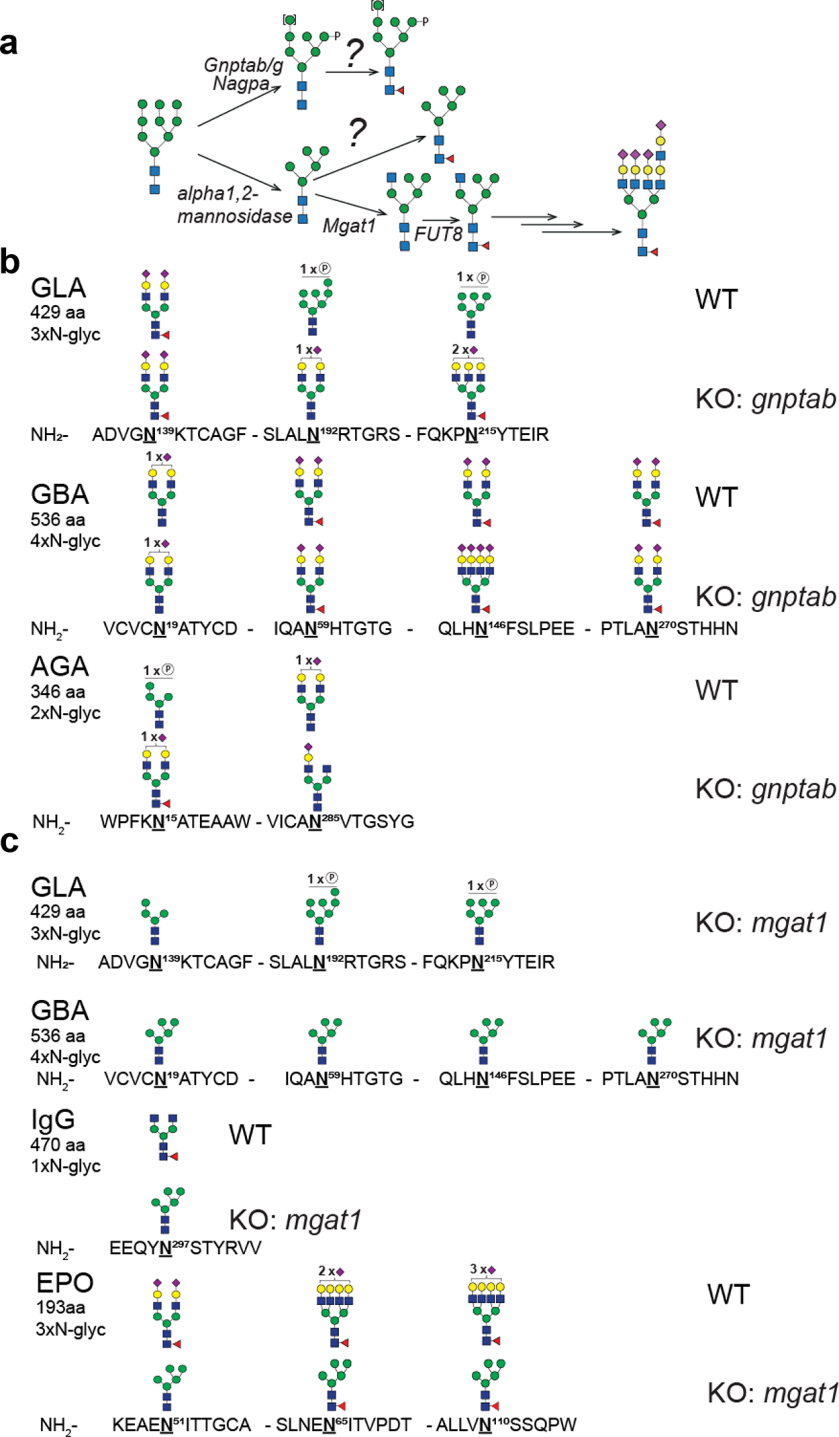
(a) Graphic depiction of genes involved in early steps of N-glycan synthesis and (b, c) *in vivo* core fucosylation of N-glycans by site-specific analysis of purified recombinant glycoproteins (IgG1, GLA, GBA, AGA, and EPO) produced in CHO^WT^ and KO of *gnptab* and *mgat1*. The most abundant glycan structures at each N-glycosite of each reporter protein produced in CHO^WT^ and engineered CHO clones are displayed. All glycan structures at each glycosite were confirmed by tandem mass spectrometry (MS/MS) analysis.

In the past, we first examined a panel of recombinant expressed secreted N-glycoprotein therapeutics studied extensively in glycoengineered CHO cells.^27^ We included anti-rabies virus human immunoglobulin IgG1, erythropoietin (EPO), and three lysosomal replacement enzymes, namely, glucocerebrosidase (GBA), α-galactosidase (GLA), and aspartylglucosaminidase (AGA) (Figure 4). To obtain a more global perspective of core fucosylation, we also performed knockout (KO) studies of transferases targeting a key glycosyltransferase (Mgat1) converting high mannose to a complex N-glycan and the GlcNAc-1-phosphotransferases (GNPTAB), which are responsible for tagging of M6P to a high-mannose N-glycan for lysosomal targeting (Figure 4a). We then performed site-specific analysis to monitor effects on glycosylation of the secreted purified proteins by LC–MS, and only the most abundant glycan at each site is presented in Figure 4 for demonstrating the major changes between wild-type (WT) and KO cells.

When expressed in CHO^WT^ cells, these N-glycoproteins carry distinct repertoires of N-glycans at select glycosites. The lysosomal enzymes have one or more complex-type (mainly biantennary) N-glycans with or without core Fuc and one or more M6P-tagged high-mannose glycans without core Fuc (Figure 4b). When the capacity for M6P-tagging is eliminated by the knockout of *gnptab* (KO *gnptab*), the M6P-tagged high-mannose glycans are all converted to the complex type and, interestingly, core Fuc is found at some but not all glycosites, as previously described.^27^ We then eliminated the capacity for the formation of complex N-glycans (KO *mgat1*) to convert all N-glycans to high-mannose structures to force substrate accumulation for the FUT8 enzyme (Figure 4c). N-glycans on the lysosomal enzymes and IgG1 were converted to high-mannose glycans without core Fuc. However, interestingly with EPO, the N-glycans at all three glycosites acquired core Fuc to some degree, with the N65 glycosite being almost exclusively core-fucosylated (Table 3). The basis for the observed differences in core fucosylation of high-mannose N-glycans in the lysosomal glycoproteins and EPO is currently unknown, but we note that the general processing of the N-glycans on EPO is also different and more elaborate with almost complete tetra-antennary structures. Thus, we propose that EPO and its glycosites are particularly accessible substrates for the Golgi-processing enzymes including FUT8. The finding that EPO is core-fucosylated in high-mannose N-glycans was also recently demonstrated by Wang and colleagues but not at the level of specific N-glycosites.^21^ Our results confirm that FUT8 primarily core-fucosylates complex-type N-glycans but also demonstrates that core fucosylation occurs at select N-glycosites and that high-mannose glycosites can rarely become core-fucosylated.

**Table 3:** Site-Specific N-glycan Analysis of EPO Expressed in CHO^WT^ and CHO^KO:*mgat1*^

		CHO^KO:*mgat1*^	
EPO N-glycosite	CHO^WT^^a^	Man5-GlcNAc2-Fuc/total^b^	Man5-GlcNAc2/total^b^
N51	biantennary	0.48	0.52
N65	tetra-antennary	0.90	0.10
N110	tetra-antennary	0.67	0.33

a Glycans at all three sites of EPO in CHO^WT^ are exclusively core-fucosylated.

b Total is the sum of top five glycopeptide intensities at each site by LC–MS.

### The Innermost Amino Acids around the N-glycan GlcNAc Affect Core Fucosylation

**Figure 5:**
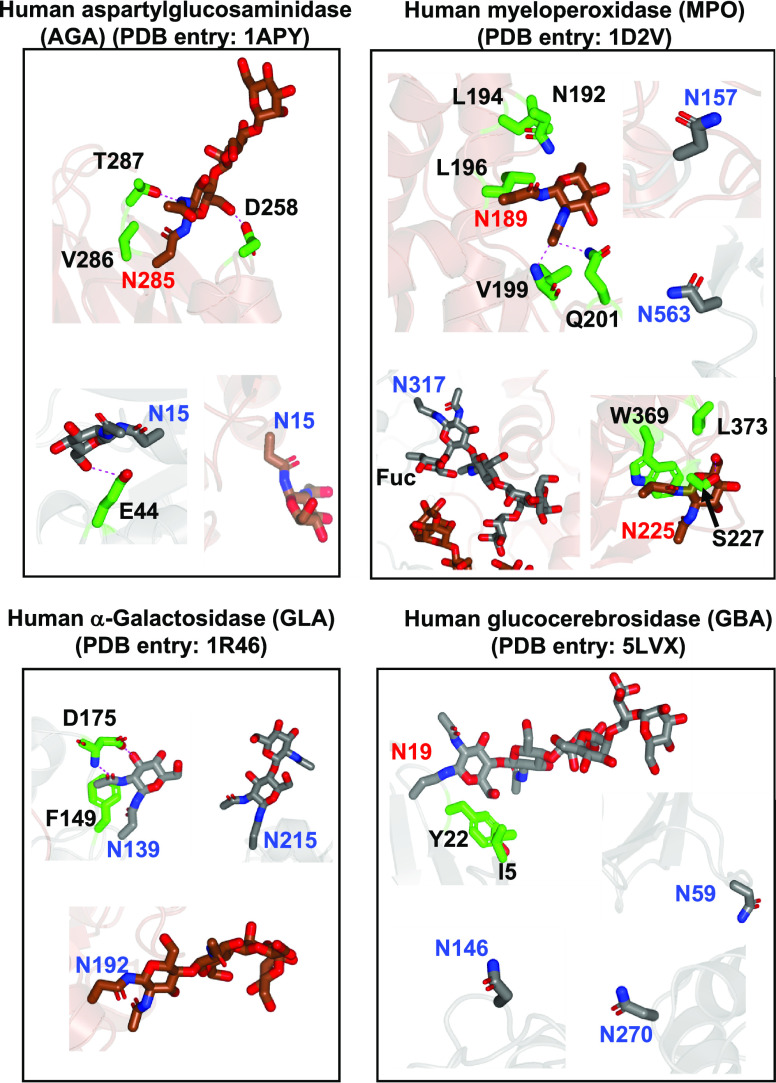
Close-up structures of the N-glycans of GBA, MPO, AGA, and GLA. Note that MPO, AGA, and GLA are dimeric structures (depicted in gray and brown colors), while GBA is monomeric (gray). Asn and residues around the N-glycans are shown as sticks with carbon atoms in gray/brown and green, respectively. Hydrogen bond interactions are shown as dotted magenta lines. Note that the residue numbering in the crystal structures for GBA, MPO, and AGA does not correspond to the numbering of the full-length proteins. For AGA, Asn15 and Asn285 from the crystal structure correspond to Asn38 and Asn308 in the full-length protein, respectively. For GBA, Asn19, Asn59, Asn146, and Asn70 correspond to Asn58, Asn98, Asn185, and Asn309, respectively. For MPO, Asn157, Asn189, Asn225, Asn317, and Asn563 correspond to Asn323, Asn355, Asn391, Asn483, and Asn729, respectively.

Our findings that the complex N-glycan at Asn19^GBA^ and Asn285^AGA^ is not core-fucosylated imply that other mechanisms other than the presence of terminal sugar moieties of the α1,6 arm or α1,3 arm are behind core fucosylation. To explore the molecular basis of this, we inspected the crystal structures of AGA, GLA, and GBA. In addition, we also inspected the human myeloperoxidase (MPO) N-glycan acceptor sites for which Asn157/Asn317/Asn563 and Asn189/Asn225 have been shown to be core-fucosylated and non-core-fucosylated, respectively (Figure 5). Most of these N-glycan acceptor sites are located in loops as typically expected for the location of N-glycans.^28^ Most of the inspected N-glycan acceptor sites that were core-fucosylated, namely, Asn59/Asn146/Asn270^GBA^, Asn157/Asn317/Asn563^MPO^, and Asn215/Asn192^GLA^, were solvent-exposed, thus allowing FUT8 access to the N-glycan and in turn core fucosylation (Figure 5). One might expect core fucosylation to occur only in the loops. However, both Asn15^AGA^ and Asn192^GLA^ are yet core-fucosylated and are located in secondary structures (Figures 4 and 5). In Asn15^AGA^, although the innermost GlcNAc OH6 was engaged in a hydrogen bond with Glu44^AGA^ from one of the monomers forming the dimer, in the other monomer, this interaction did not take place. This implies that the innermost GlcNAc OH6 would be accessible to FUT8. However, it is not entirely clear why Asn139^GLA^ is core-fucosylated because the innermost GlcNAc acetamide group and OH3 were engaged in hydrogen bond interactions with Asp175^GLA^. In addition, the acetamide methyl moiety was engaged in a CH−π interaction with Phe149^GLA^. All these interactions likely would impede core fucosylation.

All the other non-core-fucosylated N-glycan acceptor sites, Asn19^GBA^, Asn189/Asn225^MPO^, and Asn285^AGA^, are engaged in different interactions with surrounding amino acids of the protein acceptor substrates. The innermost GlcNAc moieties interact with the following residues as follows: (a) the GlcNAc moiety was engaged in a CH−π interaction with Tyr22^GBA^ and Trp369^MPO^ side chains, (b) the GlcNAc acetamide group established hydrogen bonds with the Val199^MPO^ backbone and Gln201^MPO^ and Thr287^AGA^ side chains, and (c) GlcNAc OH6 was recognized by hydrogen bonds with Asp258^AGA^ and Ser227^MPO^ side chains (Figure 5). For these particular cases, it is reasonable that they cannot be core-fucosylated as FUT8 access to the N-glycan acceptor sites is likely impeded. Further, non-interacting residues with the N-glycan acceptor sites, such as Ile5^GBA^, Asn192/Leu194/Leu196/Leu373^MPO^, and Val286^AGA^, would also likely impede core fucosylation due to steric clashes with FUT8.

Finally, we performed extensive MD simulations on the N-glycans of MPO, GLA, GBA, and EPO present in the crystal structures (see Methods). Note that for EPO, Asn24, Asn38, and Asn83 from the crystal structure correspond to Asn51, Asn65, and Asn110 in the full-length protein, respectively. In addition, in the case of the EPO protein, three Lys residues from the crystal structure (Lys24, Lys38, and Lys83), which corresponded to Asn residues in the wild-type protein, were mutated to Asn and a GlcNAc moiety was manually appended to understand how the sugar moiety might affect FUT8 binding. For MPO and GBA, we did not analyze all the N-glycans because there were no sugar moieties bound to some of the Asn residues. In general, we found the GlcNAc residues most susceptible for core fucosylation are those that show a higher solvent-accessible surface area (SASA). Thus, GlcNAc attached to Asn19^GBA^ provides a low SASA value, in good agreement with the absence of core fucosylation. On the other hand, GlcNAc residues attached to Asn317^MPO^, Asn192^GLA^, Asn139^GLA^, Asn24^EPO^, Asn38^EPO^, or Asn83^EPO^ exhibit high SASA values, explaining the tendency of these residues to undergo core fucosylation. The only exception is GlcNAc at Asn215^GLA^, which presents a low SASA value and shows a clear preference to be fucosylated (Figure S19).

We conclude that in addition to the nature of the α1,3 or α1,6 arm of the N-glycan, core fucosylation is also modulated by the environment of the N-glycan surrounding amino acids of the protein acceptor substrate. In particular, residues interacting with the innermost GlcNAc (e.g., the sugar moiety itself or specific positions such as the acetamide group or OH6) and non-interacting residues with the N-glycan can block FUT8 access to the N-glycan, impeding core fucosylation. This provides another avenue to regulate core fucosylation.

## Conclusions

Up-regulation of core fucosylation is tightly linked to cancers and is associated with poor prognosis. This PTM may also act as a “safety switch”, attenuating potentially harmful ADCC.^29^ Indeed, it has been reported that 95% of IgG N-glycans in healthy individuals have core Fuc to avoid the side effect of potent ADCC due to afucosylated antibodies.^29,30^ Recently, core fucosylation has been highly associated with the degree of symptoms of COVID-19, with critically ill COVID-19 patients having the highest levels of afucosylated IgG antibodies against SARS-CoV-2, leading to an increase in pro-inflammatory cytokine release and acute phase responses.^31^ Due to the importance of core fucosylation in physiology and disease, it is imperative that we understand FUT8’s substrate specificity, how its activity is regulated under different conditions, and how dysregulation of FUT8 relates to different pathologies.

Here, using multidisciplinary methodologies, we have inferred that two potential mechanisms are behind the acceptor substrate preferences by FUT8. In the first case, the monosaccharide residues of the α1,3/α1,6 arms determine the recognition of FUT8 on either complex N-glycans or high/low-mannose N-glycans. Although this mechanism was already proposed by others, mainly at the *in vitro* level,^11,32^ we postulate that FUT8 preferentially core-fucosylates complex N-glycans rather than high/low-mannose N-glycans *in vivo*. We believe that the second modulator of core fucosylation is driven by the nature of the residues around the N-glycosylation site, in particular, residues interacting with the peptide-linked GlcNAc via the acetamide group or OH6. These hydrogen bond interactions together with additional non-interacting residues of the acceptor protein substrate would block FUT8 binding to the N-glycans, impeding core fucosylation. It was previously suggested that the more the N-glycans are exposed to the solvent, regardless of the type of N-glycan, the more likely they will be core-fucosylated.^21^ However, no analysis of the N-glycans in the context of their protein acceptor substrates or MD simulations was performed. The latter calculations allowed us to obtain a more realistic scenario (exemplified by the SASA values) that reflect a more dynamic situation characterized by several conformations of the protein and the glycans, rather than only the conformation found in the crystal structure. Consequently, the absence of these data in the previous analysis may complicate the analysis of why certain and potentially favorable complex N-glycans are not core-fucosylated. Although FUT8 recognizes the underlying amino acids and, in particular, the OST sequon, this recognition is likely not important in the overall core fucosylation process in complex N-glycans. In contrast, we found that peptide recognition is key for core fucosylation of low- and high-mannose N-glycans. Therefore, herein, we have thoroughly demonstrated that, *in vivo*, the amino acids around the N-glycan sites could play fundamental roles in modulating N-glycan core fucosylation.

In conclusion, we demonstrate that FUT8 recognizes the OST sequon, but this peptide recognition is only critical for the *in vitro* core fucosylation of low- and high-mannose N-glycopeptides. In addition, our results confirm that FUT8 primarily core-fucosylates complex-type N-glycans and rarely high-mannose glycosites. We also discovered that some complex N-glycan acceptor sites are not core-fucosylated in cells due to surrounding amino acid residues, which may interact with the innermost GlcNAc of N-glycans or sterically clash with FUT8, implying that core fucosylation occurs at select N-glycosites. Overall, our data provide new insights into N-glycan core fucosylation *in vivo*.

## Methods

### Purification of FUT8

The human FUT8 was purified as previously described.^12^

### Isothermal Titration Microcalorimetry (ITC)

ITC was performed to characterize the interaction of FUT8 with GDP, G0, the G0-peptide, the M3N2-peptide, M3N2, and M5N2-Asn. All experiments were carried out in an Auto-iTC200 (Microcal, GE Healthcare) at 25 °C. The concentration of FUT8 was 70 μM and the ligand concentration ranged from 400 μM to 1 mM. The experiments used 25 mM Tris (pH 7.5) and 150 mM NaCl for the characterization of the binding of FUT8 against GDP and G0. To characterize the binding of FUT8 against all N-glycans in the presence of an excess of GDP, the conditions were 25 mM Tris (pH 7.5), 150 mM NaCl, and 1 mM GDP. The experiments were performed in duplicate. Data integration, correction, and analysis were carried out in Origin 7 (Microcal). The data were fitted to a one-site equilibrium-binding model.

### Kinetic Analysis

Enzyme kinetics for FUT8 were determined using GDP-Glo luminescence assays (Promega). Reactions contained 100 nM FUT8 in 25 mM Tris (pH 7.5), 150 mM NaCl, and 200 μM GDP-fucose in the presence of the ligands. The concentrations of G0, the G0-peptide, the M3N2-peptide, M3N2, and M5N2-Asn ranged from 5 μM to 1 mM. To determine the kinetic parameters for GDP-fucose, we used 100 nM FUT8 in 25 mM Tris (pH 7.5), 150 mM NaCl, and variable concentrations of GDP-fucose (from 5 to 500 μM) in the presence of 500 μM of G0. Reactions were incubated for 30 min at 37 °C and stopped using 5 μL of GDP-detection reagent at a 1:1 ratio in white and opaque 384-well plates. Then, the plates were incubated in the dark for 1 h at room temperature. Subsequently, the luminance values were obtained by using a Synergy HT plate reader (Biotek).

To estimate the amount of GDP produced in the glycosyltransferase reaction, we created a GDP standard curve. The values were corrected for enzyme hydrolysis in the absence of the substrate acceptor and fitted to a nonlinear Michaelis–Menten equation in GraphPad Prism 6 software from which the *K*_m_, *k*_cat_, and *V*_max_ along with their standard deviations were obtained. All the experiments were performed in duplicate.

### Glycan and Glycopeptide Preparation

Glycans and glycopeptides were prepared from common scaffolds followed by enzymatic modifications as depicted in Figure 1 and described below.

The preparation of G0, the G0-peptide, M3N2, and the M3N2-peptide derived from SGP isolated from egg yolk was carried out. M3N2 and the M3N2-peptide were obtained through the isolation of SGP from hen’s egg yolk following a published procedure.^19^ SGP was further processed to yield the G0-peptide, sialic acids were chemically removed with acetic acid, and galactose residues were enzymatically released with β-galactosidase from *Aspergillus niger*.^33^

### SGP-Derived Peptide Isolation

Treatment of the G0-peptide with PNGaseF produced G0 and the corresponding peptide KVANKT. The peptide was purified by C18 chromatography employing a gradient from aqueous 0.1% trifluoroacetic acid (TFA) to 20% MeOH in aqueous 0.1% TFA.

### M3N2 and M3N2-Peptide Preparation

The G0-peptide and G0 were further enzymatically processed with β-*N*-acetylglucosaminidase (New England Biolabs) to remove terminal *N*-acetylglucosamine residues, producing M3N2 and the M3N2-peptide. The purification was performed by graphitized carbon chromatography employing a gradient from water to 50% MeOH.^19,34^

### M5N2-Asn Preparation from Soy Bean Agglutinin

Soy bean agglutinin was purified by affinity chromatography from soy bean flour following a previously published procedure.^35^ M5N2-Asn was obtained by soy bean agglutinin digestion with Pronase from *Streptomyces griseus* (Sigma-Aldrich) at 37 °C followed by purification on graphitized carbon and Sephadex LH-20. Man_9_GlcNAc_2_Asn was digested with α-1,2-mannosidase from *Bacteroides thetaiotaomicron* (NZYtech) to produce M5N2-Asn.^36^ Purification was done by graphitized carbon chromatography employing a gradient from water to 50% MeOH.

### Analysis of FUT8 Substrate Specificity by MALDI-TOF

Solutions of the different N-glycan structures (1 nmol) were incubated with a FUT8 enzyme (18 μM) and GDP-fucose (2 nmol) in 25 mM Tris and 150 mM NaCl (pH 7.5) (total volume of 10 μL) for 18 h at RT. The reaction mixture was analyzed by MALDI-TOF mass spectrometry employing a solution of 2,5-dihydroxybenzoic acid (DHB, 5 mg/mL in CH_3_CN:0.1% TFA, 30:70, containing 0.05% NaCl) as a matrix. The MALDI-TOF spectra were recorded on an Ultraflextreme III time-of-flight mass spectrometer equipped with a pulsed Nd:YAG laser (355 nm) and controlled by FlexControl 3.3 software (Bruker Daltonics, Bremen, Germany). The *m*/*z* range was selected according to the mass of the sample and the acquired spectra were processed with FlexAnalysis 3.3 software (Bruker Daltonics, Bremen, Germany).

### Ligand Assignment and STD NMR Method

All experiments were performed at a temperature of 283 K using a Bruker AVANCE-III 800 MHz spectrometer with a 5 mm TXI triple resonance probe with *z* axis pulse field gradients (5 mm PATXI 1H-13C/15N/D Z-GRD). The ligands, M3N2, the M3N2-peptide, the G0-peptide, and the naked peptide, had their labile protons exchanged with deuterium by dissolving in D_2_O, freeze-drying for three times, and finally dissolving at 5 mM in D_2_O. M3N2-peptide, G0-peptide, and naked peptide ^1^H-NMR and ^13^C-NMR signals were assigned using ^1^H-^13^C HSQC (hsqcedetgp), ^1^H-^13^C HSQC-TOCSY (hsqcgpmlph), TOCSY (mlevph), COSY (cosygmpfqf), and HMBC (hmbcgplpndqf). 2D-NOESY experiments were carried out for M3N2 and the M3N2-peptide using the Bruker pulse program noesygpph with mixing times (d8) of 100, 150, 200, 250, 300, 400, 600, 800, 1000, and 1500 ms. The relaxation time was set to 1 s. Fourier transform was applied to both dimensions and the corresponding horizontal traces were extracted for H1B (4.47 ppm) and H5A (3.42 ppm) in the case of the M3N2-peptide. The phase and baseline were corrected for each trace, and the corresponding regions were integrated. Integral values were represented vs mixing time and the linear initial part of each curve was adjusted to a line to calculate the corresponding slope (Figure S9a,b). Average H1A-H5A and H1B-H5B distances were calculated from the 1 μs MD simulation and used for the calculation according to the equation in Figure S9c.

### STD NMR Measurements

STD NMR spectra were measured for the corresponding N-glycans in the presence of 20 μM FUT8 both with and without GDP (1 mM) using the Bruker pulse program stddiff.3. These experiments were performed in a buffer of 50 mM deuterated Tris and 150 mM NaCl (pH 7.4) in D_2_O. A train of 50 ms Gaussian pulses was used at 0.2 mW applied on the F2 channel at −0.5 ppm (on resonance) and 40 ppm (off resonance) to obtain the STD spectra. To remove any unwanted XY magnetization from the previous scan, a spoil sequence of two trim pulses of 2.5 and 5 ms with a 40% z-gradient applied for 3 ms at the start of the experiment was used. To suppress protein signals, a spinlock of 1.55 W and 40 ms was used (stddiff.3). To acquire build-up curves for each of the systems, the experiments were repeated with saturation times (D20) of 0.5, 1, 1.5, 2, 3, 4, and 5 s with recycle delays D1 set to 2, 2.5, 3, 3.5, 4.5, 5.5, and 6.5 s, respectively.

### Stable Expression of Recombinant Human IgG1, AGA, GLA, and GBA in CHO Cells

CHOZN GS–/– cells (Merck) were maintained in suspension cultures in serum-free media (EX-CELL CHO CD Fusion, cat. no. 14365C), supplemented with 4 mM l-glutamine as previously described.^37^ An expression construct containing anti-rabies human IgG1^37^ was used to establish stably expressing CHO clones as reported before. The entire coding sequence of human GLA, GBA, and AGA was synthesized by Genewiz, USA. All reporter constructs were cloned into pCGS3 (Merck). Cells were seeded at 0.5 × 10^6^ cells/mL in a T25 flask (NUNC, Denmark) 24 h prior to transfection. A total of 2 × 10^6^ cells were transfected with 8 μg of endotoxin-free plasmids using the Amaxa kit V and program U24 with Amaxa Nucleofector 2B (Lonza, Switzerland). The 72 h post-transfection cells were plated at 500–1000 cells/well in 96-well plates in 200 μL of Minipool Plating Medium containing an 80% EX-CELL CHO Cloning Medium (cat. no. C6366) and 20% EX-CELL CD CHO Fusion medium without glutamine for selection. Screenings of high expression clones were performed by enzyme activity assay using a medium for AGA or anti-hFc-HRP antibody (Merck). Selected clones were expanded in 50 mL TPP TubeSpin shaking Bioreactors (180 rpm, 37 °C, and 5% CO_2_).

### Purification of Recombinant Reporter Proteins Expressed in CHO Cells

Culture media were centrifuged at 500*g* for 20 min and filtered (0.45 μm). Purification of IgG1 and GLA, GBA, and EPO was performed as reported in previous works.^27,37,38^ For AGA, 20% (v/v) conditioning buffer (70 mM Tris–HCl, pH 7.0) was added to the media and loaded on a column packed with Q-FastFlow Sepharose (GE Healthcare) pre-equilibrated with 5 column volumes (CV) of equilibration buffer (20 mM Tris–HCl, 20 mM sodium acetate, 70 mM sodium chloride, pH 6.8). After washing the column with 6 CV of wash buffer (20 mM Tris–HCl, 20 mM sodium acetate, 70 mM sodium chloride, pH 6.8), the enzyme was one-step eluted with elution buffer (25 mM sodium acetate, 250 mM NaCl, pH 4.5) into a tube containing 300 mM sodium phosphate (pH 7.3). The eluates were diluted with 50% (v/v) 4 M (NH_4_)_2_SO_4_ and further loaded on a Phenyl-Sepharose Fast Flow (high substitution) column (GE Healthcare). After washing and equilibrating the column with 5 CV of 2 M (NH_4_)_2_SO_4_ and 20 mM Tris–HCl (pH 7.0), the enzyme was eluted with elution buffer in a gradient (from 2 M to 0 M (NH_4_)_2_SO_4_, 20 mM Tris–HCl, pH 7.0).

### CRISPR/Cas9-Targeted KO in CHO Cells

Gene targeting was carried out in CHO clones with stable expression of reporter proteins. Cells were seeded at 0.5 × 10^6^ cells/mL in a T25 flask (NUNC, Denmark) 24 h prior to transfection, and 2 × 10^6^ cells and 1 μg each of plasmid DNA of Cas9-GFP and gRNA were used for electroporation. Forty-eight hours after electroporation, cells with GFP expression were enriched by FACS. After culturing for 1 week, cells were single cell-sorted by FACS into 96-well plates. KO clones with desired mutations were screened by a fast screening and selection method, i.e., Indel Detection by Amplicon Analysis (IDAA) as described.^39^ Final clones were verified by Sanger sequencing. On average, two to five clones were selected from each targeting event with frameshift mutations. The full list of CRISPR gRNA design and PCR primers used is listed elsewhere.^37^

### Sample Preparation for N-glycopeptide Site-Specific Analysis

Twenty-five micrograms of purified GLA, GBA, and AGA was dissolved in 50 mM ammonium bicarbonate (AmBic) buffer (pH 7.4) and further reduced with 10 mM dithiothreitol (DTT) at 60 °C for 45 min on a shaker, followed by alkylation with 20 mM iodoacetamide (IAA) at 25 °C for 30 min in the darkness. The sample was proteolytically digested with chymotrypsin (1:40 enzyme/substrate ratio). The reaction was quenched with 1 μL of TFA and the digested sample was desalted by custom-made modified StageTip columns with three layers of C18 and two layers of C8 membrane (3 M Empore disks, Sigma-Aldrich). Samples were eluted with two steps of 50 μL of 50% methanol in 0.1% formic acid. The final sample was aliquoted in two equal parts. The first aliquot was placed into a glass insert (Agilent, USA), dried completely in a SpeedVac (Eppendorf, Germany), further redissolved in 50 μL of 0.1% formic acid (FA), and submitted for nLC-MS analysis. The second aliquot was placed in an Eppendorf tube, dried completely in a SpeedVac, and redissolved in 50 μL of 50 mM AmBic buffer (pH 7.4), followed by addition of 1 U PNGase F per sample at 37 °C for 12 h on a shaker. The sample treated with PNGase F was desalted, dried by the same methods mentioned above for the first aliquot, and submitted for nLC-MS/MS analysis.

### IgG N-glycan Analysis by CE-LIF

The analysis of IgG1 N-glycans was also performed by capillary electrophoresis as described below.^40^ Briefly, 15 μL of Protein G Sepharose beads (GE Healthcare) was mixed with 200 μL of conditioned medium in a 96-well plate. After washing two times with 50 mM NH_4_HCO_3_, 1 U PNGaseF (Roche) was added and the plate was incubated for 1 h at 50 °C. The reaction mixture was then adjusted to 87.5% ACN and mixed with 15 μL of pre-equilibrated carboxyl-coated magnetic beads (Thermo Scientific). Beads were washed twice with 87.5% ACN on a 96-well magnet stand, mixed with 6 μL of 40 mM APTS in 20% acetic acid and 2 μL of 1 M NaBH_3_CN in tetrahydrofuran, and incubated for 2 h at 37 °C. After the labeling, excess APTS was removed by washing twice with 87.5% ACN on the glycan-absorbed magnetic beads, and the labeled N-glycans were released in 40 μL of MQ water. For capillary electrophoresis analysis, 2 μL of the labeled N-glycan was mixed with 8 μL of HiDi Formamide and 0.05 μL of 500GS-LAS standard and injected into a genetic analyzer equipped with 24-capillary and laser-induced fluorescence detection (LIF, Thermo Fisher 3500 × l). Data were analyzed using GeneMapper software.

### nLC-MS/MS Analysis of Glycans and Glycopeptides

An EASY-nLC 1200 LC system (Thermo Fisher Scientific) interfaced via a nanoSpray Flex ion source to an Orbitrap Fusion Lumos MS (Thermo Fisher Scientific) was used for MS and MS/MS analysis. A single analytical column setup using PicoFrit Emitters (New Objectives, 75 μm in inner diameter) custom-packed with a Reprosil-Pure-AQ C18 phase (1.9 μm in particle size and 19–21 cm in column length) was applied in nLC. Two microliters of each sample was injected onto the column, followed by elution with a gradient of solvent B from 3 to 32% at 200 nL/min for 45 min (solvent A: 100% H_2_O + 0.1% (v/v) formic acid; solvent B: 80% acetonitrile + 0.1% (v/v) formic acid). With the nominal resolution setting of 120,000, precursors of MS1 scan (*m/z* 350–2000) were obtained. Then, HCD-MS2 of the five most abundant multiply charged precursors in the MS1 spectrum was acquired at the nominal resolution setting of 120,000. To trigger data-dependent fragmentation events, the minimum MS1 signal threshold was 50,000. Targeted MS/MS analysis was performed by setting up a targeted MSn (tMSn) scan.

### Data Analysis

Glycopeptide compositional analysis was obtained from *m*/*z* features using in-house-written SysBioWare software. For *m*/*z* feature recognition from full MS scans, Minora Feature Detector Node of the Proteome Discoverer 2.2 (Thermo Fisher Scientific) was used. A list of precursor ions (*m/z*, charge and retention time) was imported as ASCII data into SysBioWare and compositional assignment within 5 ppm mass tolerance was performed. The main building blocks used for the compositional analysis were NeuAc, Hex, HexNAc, dHex, and phosphate.

For N-glycopeptide compositional analysis, the most prominent peptides corresponding to each potential glycosite were determined experimentally by comparing the yield of deamidated peptides before and after PNGase F treatment and also added as a building block for the compositional assignment. The peptide sequence was determined by HCD MS/MS and the abundance level was calculated from PD 2.2. A list of potential glycans and glycopeptides for each glycosite was generated and the top 10–15 most abundant candidates were selected for targeted MS/MS analysis to confirm the proposed structure. Each targeted MS/MS spectrum was subjected to manual interpretation. Since the same N-glycan composition may represent isobaric structures, the listed glycan structures were made to agree with literature data. Note that the same N-glycan composition may represent isobaric structures, so the assignments of the listed glycan structures were made to agree with the predicted enzyme functions of the targeted genes together with fragmentation information in the MS/MS spectra.

### Molecular Dynamics (MD) Simulations

MD simulations were performed using AMBER 20 implemented with the ff14SB and GLYCAM06j-1 force fields. The 3D models of carbohydrate M3N2 and glycopeptides M3N2-peptide and G0-peptide were generated using GLYCAM-Web (http://glycam.org). The X-ray structures of FUT8 (PDB entry: 6TKV), MPO (PDB ID: 1D2V), GLA (PDB ID: 1R46), GBA (PDB ID: 5LVX), and EPO (PDB entry: 1CN4) were used to simulate these proteins. The setup for the MD simulations was similar to that described previously by Compañón *et al.*(41)

## Supporting Information Available

Additional methodology and figures (19 figures) for results and discussion (PDF)
