# Supplementary material for: FUT8-Directed Core Fucosylation of N-glycans Is Regulated by the Glycan Structure and Protein Environment
Source: ACS Catal. 2021 Jul 8;11(15):9052–65. doi: 10.1021/acscatal.1c01698 (PMC9161449; doi:10.1021/acscatal.1c01698)
Supplement: Supplementary file 1 — cs1c01698_si_001.pdf [file cs1c01698_si_001.pdf]

SUPPORTING INFORMATION

FUT8-directed core fucosylation of N-glycans is regulated by the glycan structure and protein environment

Ana García-García[1], Sonia Serna[2,3], Zhang Yang[4], Ignacio Delso[5], Víctor Taleb[1], Thomas Hicks[5], Raik Artschwager[2,3], Sergey Y. Vakhrushev[4], Henrik Clausen[4], Jesús Angulo[5,6,7], Francisco Corzana[8], Niels C. Reichardt[2,3,9] and Ramon Hurtado-Guerrero[1,4,10*]

[1]Institute of Biocomputation and Physics of Complex Systems (BIFI), University of Zaragoza, Mariano Esquillor s/n, Campus Rio Ebro, Edificio I+D, Zaragoza 50018, Spain

[2]CIC biomaGUNE, Paseo Miramón 182, San Sebastián 20014, Spain

[3]Basque Research and Technology Alliance (BRTA), Paseo Miramón 182, San Sebastian 20850, Spain

[4]Copenhagen Center for Glycomics, Department of Cellular and Molecular Medicine, University of Copenhagen, Copenhagen DK-2200, Denmark

[5]School of Pharmacy, University of East Anglia, Norwich Research Park, Norwich, NR4 7TJ, UK

[6]Departamento de Química Orgánica, Universidad de Sevilla, 41012, Sevilla 41012, Spain

[7] Instituto de Investigaciones Químicas (CSIC-US). Avda. Américo Vespucio 49, Sevilla 41092, Spain

[8]Departamento de Química, Universidad de La Rioja, Centro de Investigación en Síntesis Química, E-26006, Logroño, Spain

[9]CIBER-BBN, Paseo Miramón 182, San Sebastian 20014, Spain

[10]Fundación ARAID, Zaragoza 50018, Spain

^These authors contributed equally: Ana García-García, Sonia Serna, Zhang Yang and Ignacio Delso

[*]To whom correspondence should be addressed: rhurtado@bifi.es

Contents

Methods:

- SGP derived peptide (KVANKT) NMR data
- M3N2-peptide NMR data
- G0-peptide NMR data

Figures

Methods

SGP derived peptide (KVANKT) NMR data

^{1}H NMR (500 MHz, D_2O) δ 4.68 (dd, J = 7.7, 5.8 Hz, 1H), 4.46 – 4.35 (m, 3H), 4.32 (q, J = 7.2 Hz, 1H), 4.15 (d, J = 7.6 Hz, 1H), 4.08 (t, J = 6.6 Hz, 1H), 3.10 – 2.96 (m, 4H), 2.91 (dd, J = 16.9, 5.8 Hz, 1H), 2.81 (dd, J = 16.9, 7.8 Hz, 1H), 2.07 (dq, J = 13.4, 6.7 Hz, 1H), 1.97 – 1.85 (m, 3H), 1.84 – 1.65 (m, 2H), 1.55 – 1.34 (m, 5H), 1.19 (d, J = 6.4 Hz, 3H), 0.97 (d, J = 6.7 Hz, 6H).

M3N2-peptide NMR data

^{1}H-NMR (D_2O, 800 MHz) δ 4.97-4.96 (m, 1H, H1-D), 4.91 (d, J = 9.7 Hz, 1H, H1-A), 4.79-4.78 (m, 1H, H1-E), 4.65 (s, 1H, H1-C), 4.53 (dd, J = 7.6, 5.8 Hz, 1H, H2-Asn), 4.48 (d, J = 8.1 Hz, 1H, H1-B), 4.27 (dd, J = 8.1, 6.5 Hz, 1H, H2-Lys2), 4.17 (q, J = 7.3 Hz, 1H, H2-Ala), 4.12 (d, J = 2.5 Hz, 1H, H2-C), 4.08 (qd, J = 6.5, 4.5 Hz, 1H, H3-Thr), 4.00 (d, J = 6.8 Hz, 1H, H2-Val), 3.99 (d, J = 3.1 Hz, 1H, H2-Thr), 3.93 (dd, J = 3.4, 1.8 Hz, 1H, H2-D), 3.85 (dd, J = 3.4, 1.7 Hz, 1H, H2-E), 3.80-3.73 (m, 7H, H2-Lys1, H3-D, H3-E, H6-B, H6-C, H6-D, H6-E), 3.73-3.65 (m, 5H, H2-A, H2-B, H5-D, H6-A, H6-C), 3.65-3.56 (m, 8H, H3-A, H3-B, H3-C, H4-B, H4-C, H6-B, H6-D, H6-E), 3.54-3.46 (m, 7H, H4-A, H4-D, H4-E, H5-B, H5-C, H5-E, H6-A), 3.43 (ddd, J = 9.9, 4.5, 2.2 Hz, 1H, H5-A), 2.88-2.85 (m, 4H, H6-Lys1, H6-Lys2), 2.73 (dd, J = 16.5, 5.8 Hz, 1H, H3-Asn), 2.62 (dd, J = 16.5, 7.9 Hz, 1H, H3-Asn), 1.94 (s, 3H, HAc-B), 1.94-1.90 (m, 1H, H3-Val), 1.87 (s, 3H, HAc-A), 1.79-60 (m, 4H, H3-Lys1, H3-Lys2), 1.60-1.53 (m, 4H, H5-Lys1, H5-Lys2), 1.36-1.26 (m, 4H, H4-Lys1, H4-Lys2), 1.25 (d, J = 7.2 Hz, 3H, H3-Ala), 1.04 (d, J = 6.4 Hz, 3H, H4-Thr), 0.84 (d, J = 6.8 Hz, 6H, H4-Val).

^{13}C-NMR (D_2O, 200 MHz) δ 176.3 (C1-Thr), 175.7 (C1-Lys1), 174.6 (CO-B), 174.6 (CO-A), 174.2 (C1-Ala), 172.9 (C1-Lys2), 172.7 (C1-Val), 172.2 (C1-Asn), 171.7 (C4-

Asn), 102.4 (C1-D), 101.2 (C1-B), 100.3 (C1-C), 99.5 (C1-E), 80.5 (C3-C), 79.6 (C4-B), 78.5 (C4-A), 78.1 (C1-A), 76.1 (C5-A), 74.4 (C5-B), 74.1 (C5-C), 73.4 (C5-D), 72.8 (C3-A), 72.6 (C5-E), 71.9 (C3-B), 70.3 (2C, C3-D, C3-E), 70.1 (C2-C), 69.9 (C2-D), 69.7 (C2-E), 67.9 (C3-Thr), 66.7 (2C, C4-D, C4-E), 65.8 (C6-C), 65.7 (C4-C), 61.0 (C6-D), 60.9 (C6-E), 60.6 (C2-Thr), 59.9 (C6-B), 59.8 (C6-A), 59.4 (C2-Val), 54.7 (C2-B), 53.6 (C2-A), 53.5 (C2-Lys2), 53.0 (C2-Lys1), 49.9 (C2-Asn), 49.5 (C2-Ala), 39.1 (2C, C6-Lys1, C6-Lys2), 36.4 (C3-Asn), 31.5 (C3-Lys1), 30.5 (C3-Lys2), 30.0 (C3-Val), 26.3 (2C, C5-Lys1, C5-Lys2), 22.2 (CH_3-B), 22.0 (CH_3-A), 21.8 (C4-Lys2), 21.4 (C4-Lys1), 19.2 (CH_3-Thr), 18.4 (CH_3-Val), 17.7 (CH_3-Val), 16.6 (CH_3-Ala).

G0-peptide NMR data

^{1}H-NMR (D_2O, 800 MHz) δ 4.98 (s, 1H, H1-D), 4.91 (d, J = 9.7 Hz, 1H, H1-A), 4.78 (s, 1H, H1-F), 4.64 (s, 1H, H1-C), 4.54 (t, *J* = 6.9 Hz, 1H, H2-Asn), 4.47 (d, *J* = 8.1 Hz, 1H, H1-B); 4.42 (d, *J* = 8.4 Hz, 2H, H1-E, H1-G), 4.27 (dd, *J* = 8.1, 6.6 Hz, 1H, H2-Lys2), 4.23 (d, *J* = 3.5 Hz, 1H, H2-Thr), 4.23-4.19 (m, 2H, H3-Thr), 4.17 (q, *J* = 7.2 Hz, 1H, H2-Ala), 4.12 (s, 1H, H2-C); 4.06-4.04 (m, 1H, H2-D), 4.02 (d, *J* = 7.7 Hz, 1H, H1-E); 3.98-3.96 (m, 1H, H2-F), 3.94 (t, *J* = 6.7 Hz, 1H, H2-Lys1), 3.84-3.72 (m, 8H, H3-D, H3-F, H6-B, H6-C, H6-D, H6-E, H6-F, H6-G), 3.72-3.67 (m, 2H, H2-A, H6-A), 3.67-3.59 (m, 11H, H2-B, H3-A, H3-B, H3-C, H4-B, H4-C, H5-D, H6-B, H6-C, H6-E, H6-G), 3.59-3.54 (m, 2H, H2-E, H2-G), 3.54-3.45 (m, 7H, H4-A, H5-B, H5-C, H5-D, H6-A, H6-D, H6-F), 3.44-3.40 (m, 3H, H3-E, H3-G, H5-A), 3.40-3.27 (m, 6H, H4-D, H4-E, H4-F, H4-G, H5-E, H5-G), 2.89-2.85 (m, 4H, H6-Lys1, H6-Lys2), 2.72 (dd, *J* = 16.6, 6.1 Hz, 1H, H3-Asn), 2.60 (dd, *J* = 16.5, 7.5 Hz, 1H, H3-Asn), 1.94 (s, 3H, HAc-B), 1.94-1.91 (m, 1H, H3-Val), 1.92 (s, 3H, HAc-E), 1.92 (s, 3H, HAc-G), 1.87 (s, 3H, HAc-A), 1.80-1.72 (m, 3H, H3-Lys1, H3-Lys2), 1.67-1.11 (m, 1H, H3-Lys2), 1.60-1.53 (m, 4H, H5-

Lys1, H5-Lys2), 1.36-1.26 (m, 4H, H4-Lys1, H4-Lys2), 1.25 (d, *J* = 7.1 Hz, 3H, H3-Ala), 1.06 (d, *J* = 6.4 Hz, 3H, H4-Thr), 0.83 (d, *J* = 6.8 Hz, 6H, H4-Val).

^{13}C-NMR (D_2O, 200 MHz) δ 174.7 (2C, CO-E, CO-G), 174.7 (CO-A), 174.6 (CO-B), 174.3 (C1-Thr), 174.2 (C1-Ala), 173.5 (C1-Lys2), 172.5 (C1-Val), 172.1 (C1-Asn), 172.1 (C4-Asn), 169.5 (C1-Lys1), 101.2 (C1-C), 100.3 (C1-C), 99.5 (C1-D), 99.5 (2C, C1-E, C1-G), 96.8 (C1-F), 80.4 (C3-C), 79.5 (C4-B), 78.6 (C4-A), 78.1 (C1-A), 76.5 (C2-D), 76.2 (C2-F), 76.1 (C5-A), 75.7 (2C, C5-E, C5-G), 74.3 (C5-B), 74.3 (C5-C), 73.5 (C5-D), 73.3 (2C, C3-E, C3-G), 72.8 (C5-F), 72.7 (C3-A), 71.8 (C3-B), 70.1 (C2-C), 69.8 (2C, C4-E, C4-G), 69.3 (2C, C3-D, C3-F), 67.4 (C3-Thr), 67.2 (2C, C4-D, C4-F), 67.2 (2C, C3-D, C3-F), 65.9 (C6-C), 65.5 (C4-C), 61.6 (2C, C6-D, C6-F), 60.5 (2C, C6-E, C6-G), 59.8 (C6-B), 59.8 (C6-A), 59.4 (C2-Val), 58.8 (C2-Thr), 55.3 (2C, C2-E, C3-G), 54.8 (C2-B), 53.6 (C2-A), 53.5 (C2-Lys2), 52.6 (C2-Lys1), 49.9 (C2-Asn), 49.4 (C2-Ala), 39.0 (2C, C6-Lys1, C6-Lys2), 36.4 (C3-Asn), 30.4 (2C, C3-Lys1, C3-Lys2), 30.1 (C3-Val), 26.3 (2C, C5-Lys1, C5-Lys2), 22.3 (CH_3-E), 22.3 (CH_3-G), 22.2 (CH_3-B), 22.0 (CH_3-A), 21.9 (C4-Lys2), 21.1 (C4-Lys1), 19.0 (CH_3-Thr), 18.4 (CH_3-Val), 17.8 (CH_3-Val), 16.7 (CH_3-Ala).

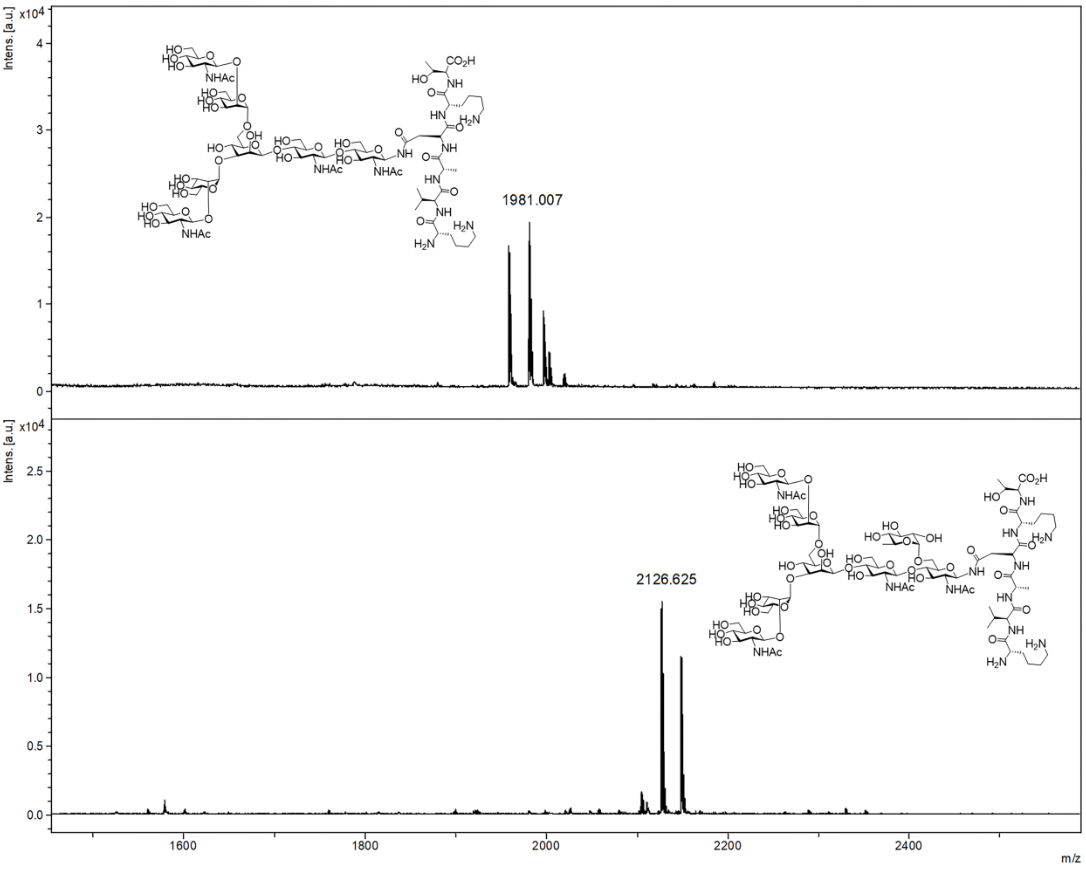

Figure S1. MALDI-tof spectra of the G0-peptide $[M+Na]^{+}$=1981.007 (upper panel) and reaction of the G0-peptide with FUT8 in the presence of GDP-Fuc (lower panel) showing complete conversion to the core fucosylated glycopeptide $[M+Na]^{+}$=2126.625 after 18 h of incubation.

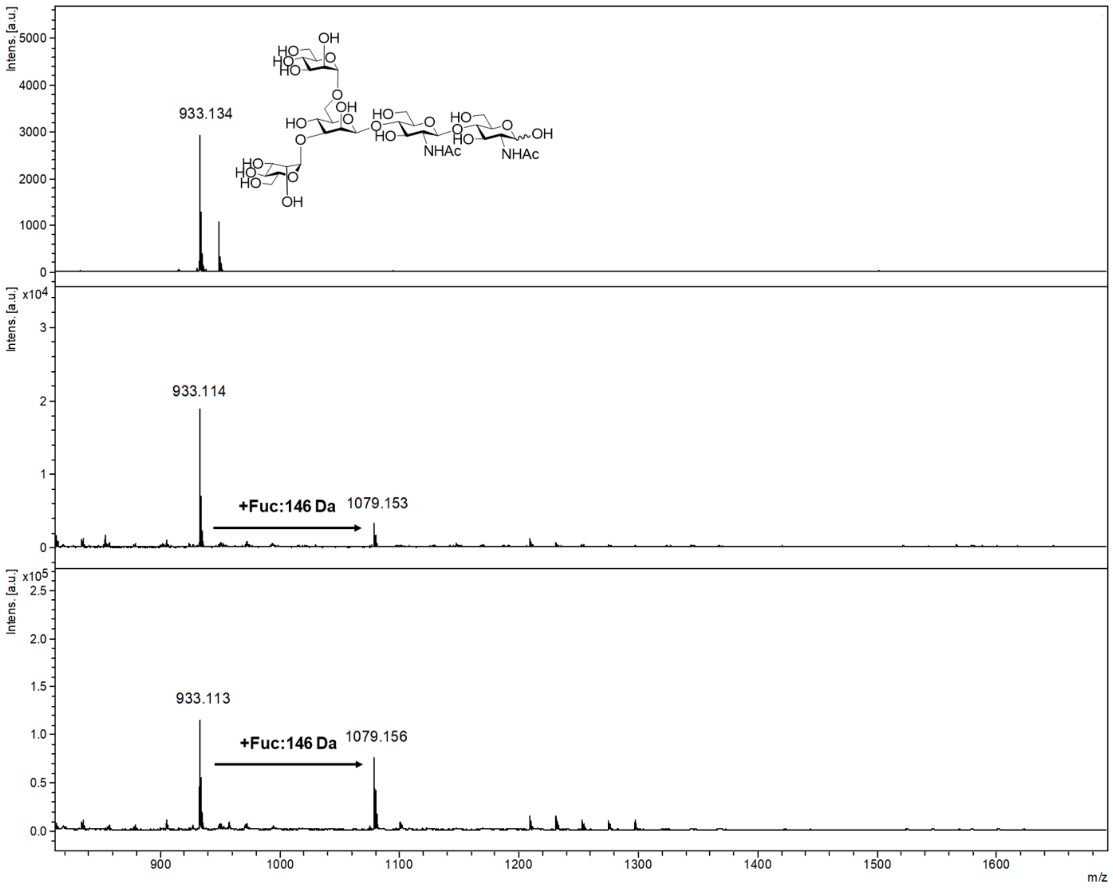

Figure S2. MALDI-tof spectra of the M3N2 $[M+Na]^+$=933.134 (upper panel) and reaction of the M3N2 with FUT8 in the presence of GDP-Fuc showing partial conversion to the core fucosylated M3N2 N-glycan $[M+Na]^+$=1079.15 after 18 h (middle panel) and 114 h (lower panel) of incubation.

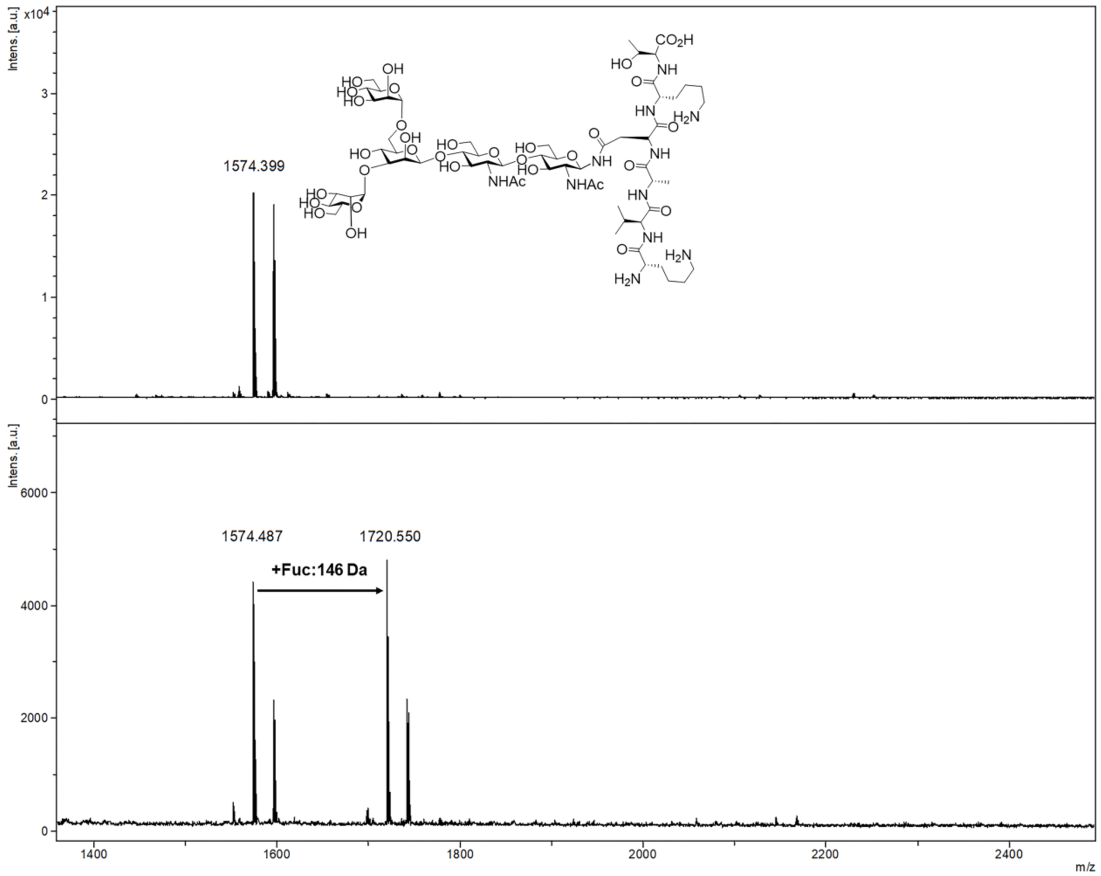

Figure S3. MALDI-tof spectra of the M3N2-peptide $[M+Na]^+$=1574.399 (upper panel) and reaction of the M3N2-peptide with FUT8 in the presence of GDP-Fuc (lower panel) showing partial conversion to the core fucosylated glycopeptide $[M+Na]^+$=1720.550 after 18 h of incubation.

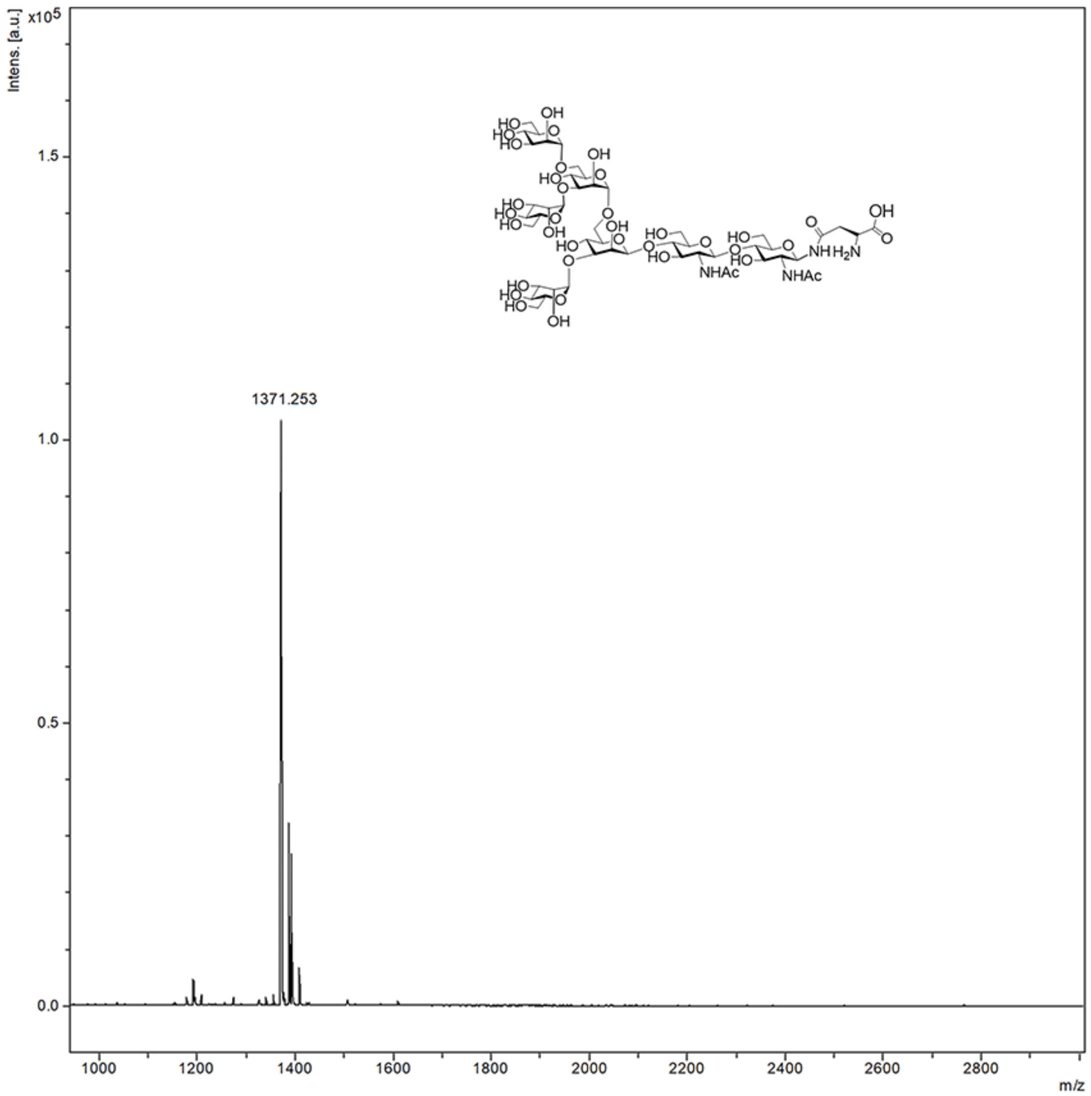

Figure S4. MALDI-tof spectrum of the M5N2-Asn $[M+Na]^+$=1371.253.

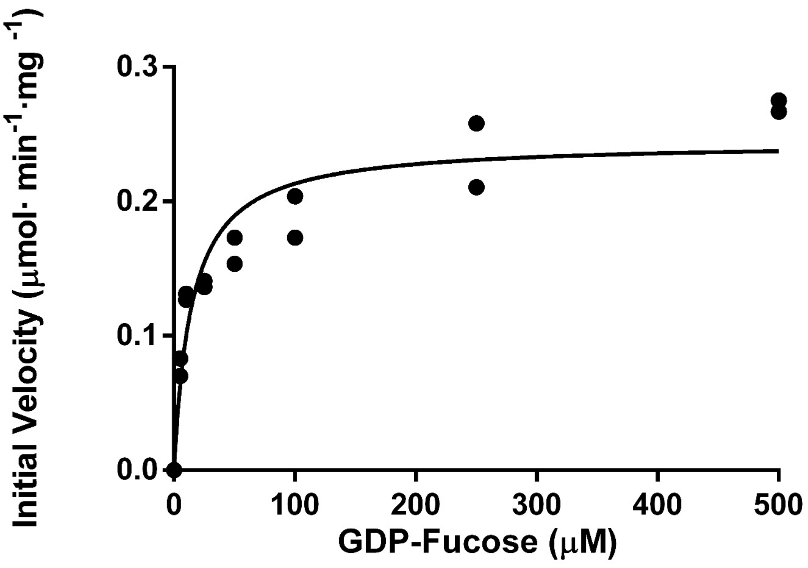

Figure S5. Kinetics of FUT8 against variable concentrations of GDP-Fuc using 500 μM of G0.

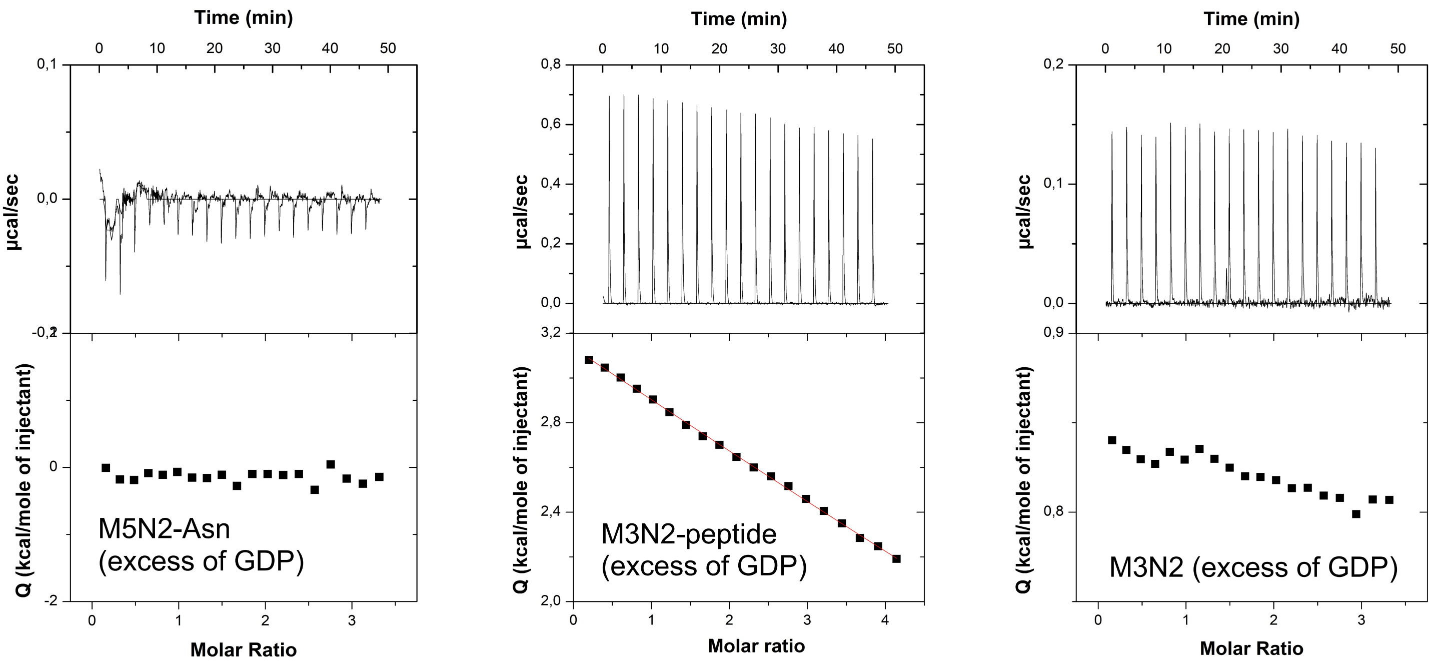

Figure S6. ITC data for the binding of the M5N2-Asn, the M3N2-peptide and the M3N2 binding to FUT8 under an excess of GDP (1 mM). Top: raw thermogram (thermal power versus time). Bottom: binding isotherm (normalized heats versus molar ratio). See Table 2 for the thermodynamic and K_d values for these experiments.

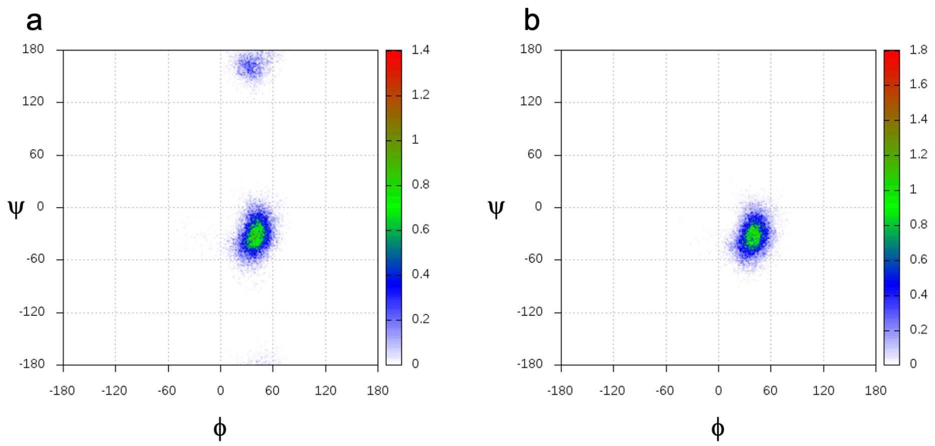

Figure S7. Molecular dynamic simulation of M3N2-peptide. ϕ/ψ angles distribution derived from 1 μs MD simulation of M3N2-peptide starting from the *anti* conformer (a) and *syn* conformer (b). Dihedral angle definition ϕ = H1(B)–C1–(B)–O4(A)–C4(A), ψ = C1–(B)–O4(A)–C4(A)–H4.

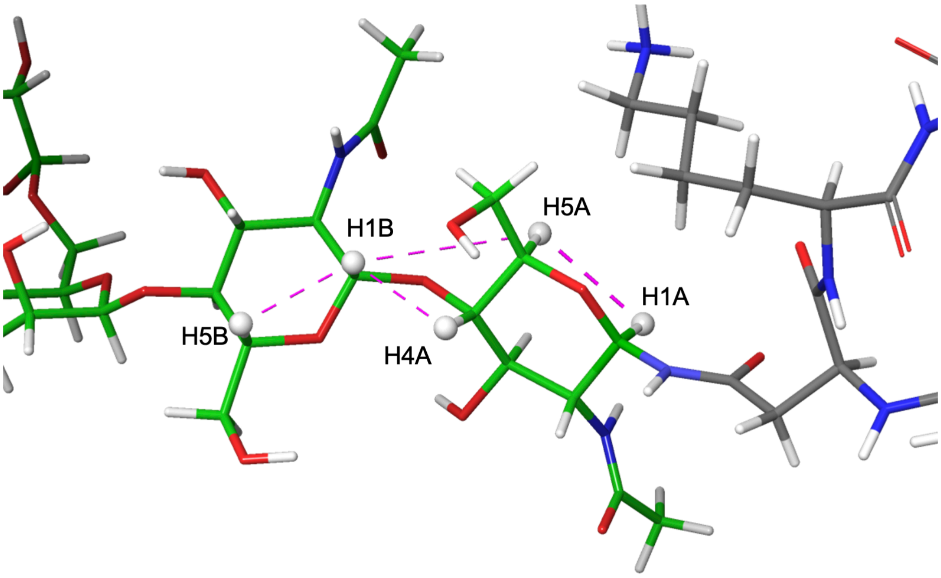

Figure S8. H1B–H4A and H1B–H5A distances were considered for the assignment of the *syn* or *anti* conformation of the glycosidic linkage between the GlcNAc moieties. Distances H1A–H5A and H1B–H5B are used as a reference.

a

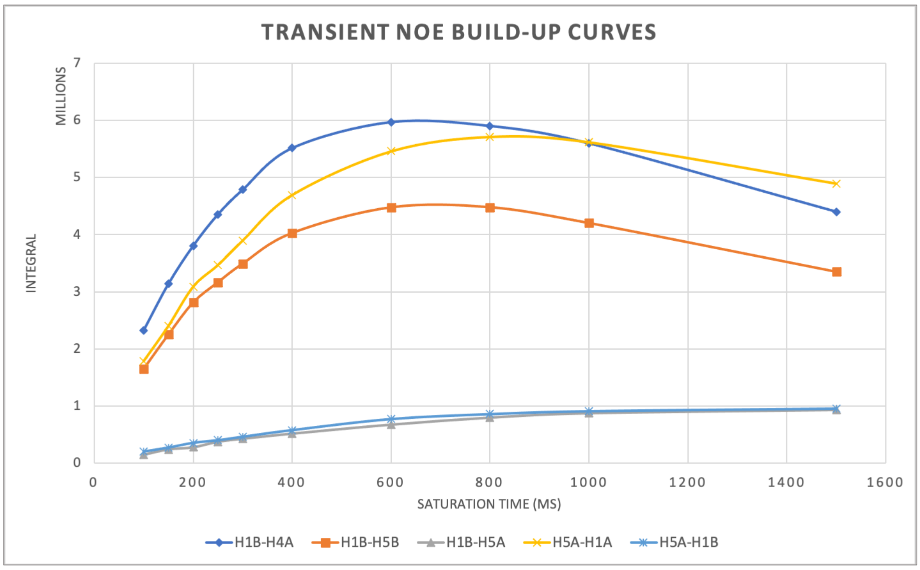

b

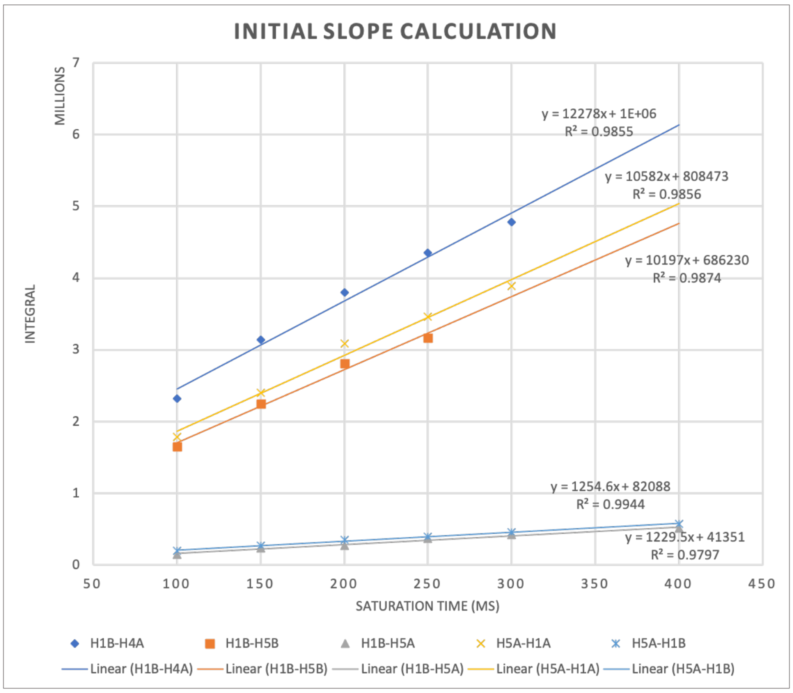

c

$$\frac{\sigma_{ij}}{\sigma_{ki}} = \frac{d_{ki}^6}{d_{ij}^6}$$

Figure S9. NOESY measurements for M3N2-peptide. a) NOE build-up curves for selected proton pairs, b) initial slope (σ) calculation and c) equation employed for distance (d) calculation.

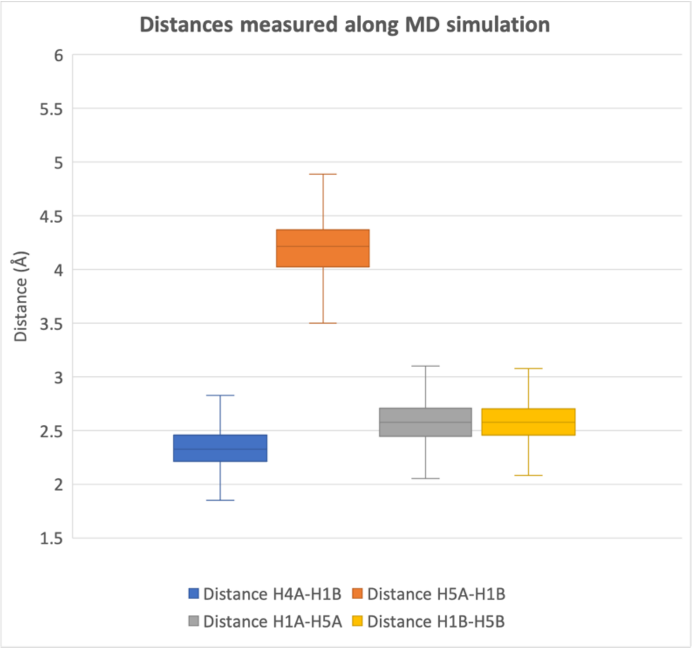

Figure S10. Molecular dynamic simulation of Man3-peptide. Interatomic distances measured along 1 µs MD simulation of Man3-peptide starting from the *syn* conformer. Distances H1A-H5A and H1B-H5B were extracted as reference distances for inter-glycosidic distance calculation.

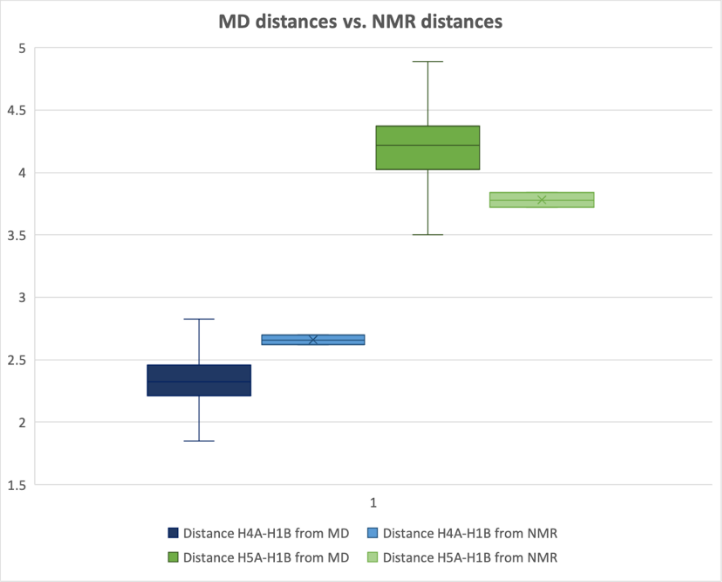

Figure S11. M3N2-peptide average conformation determination. Comparison of distances for H4A-H1B (blue) and H5A-H1B (green) measured from *syn* conformer MD simulation (dark) and calculated from NMR data (light green).

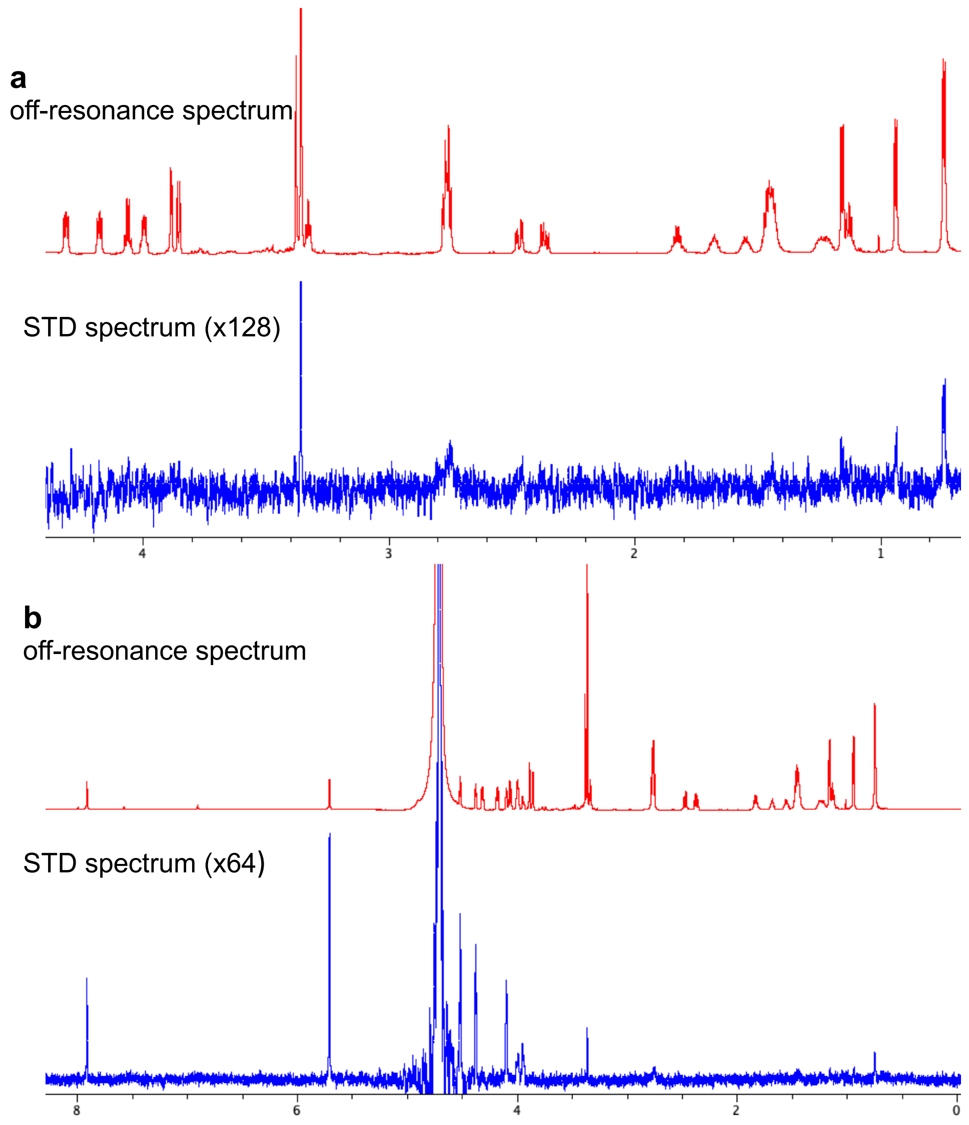

Figure S12. STD NMR analysis spectra for the naked peptide (KVANKT) in complex with FUT8 in the absence (a) and presence (b) of GDP. STD NMR spectra are scaled as indicated. Protein saturation was achieved by irradiation at -0.50 ppm.

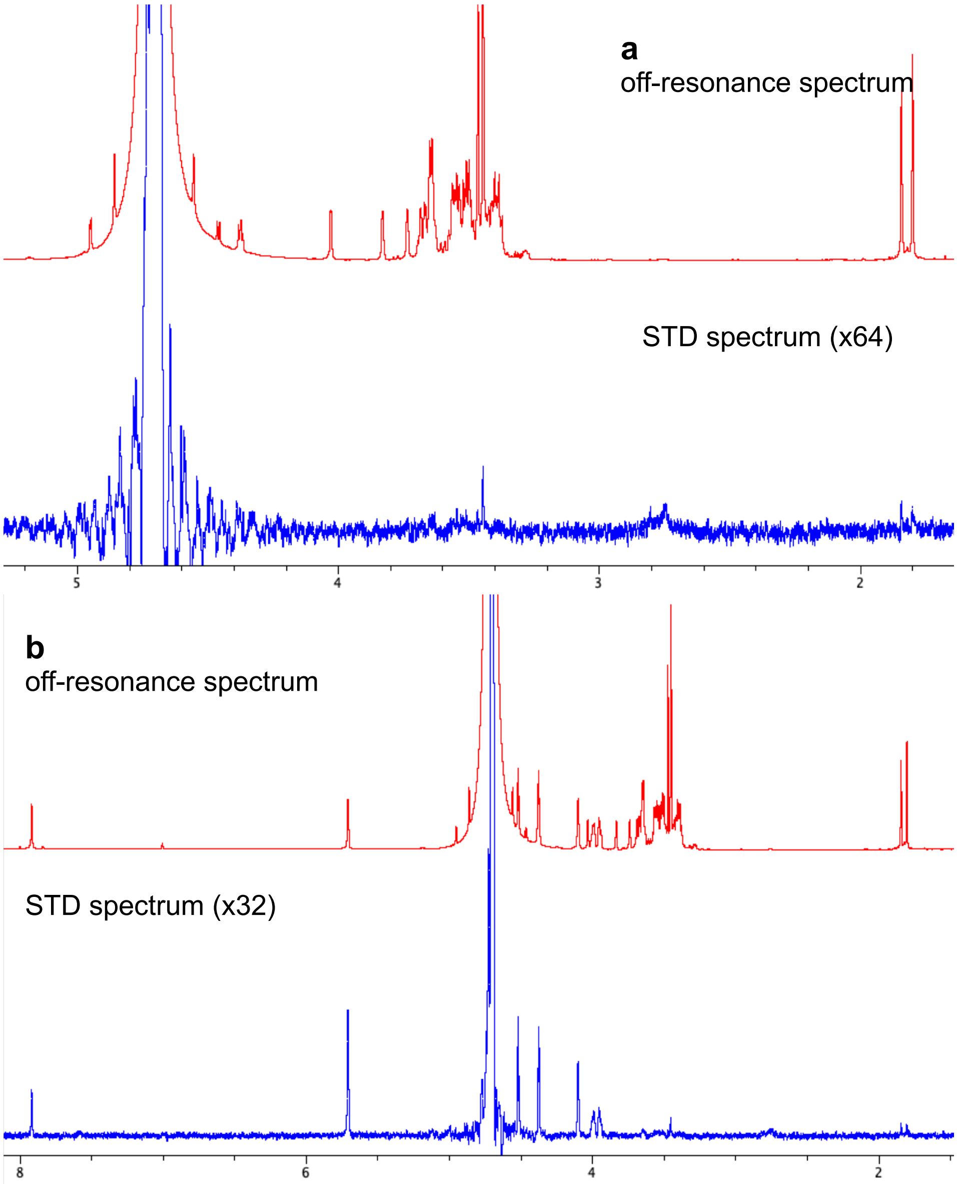

Figure S13. STD NMR analysis spectra for the M3N2 in complex with FUT8 in the absence (a) and presence (b) of GDP. STD NMR spectra are scaled as indicated. Protein saturation was achieved by irradiation at -0.50 ppm.

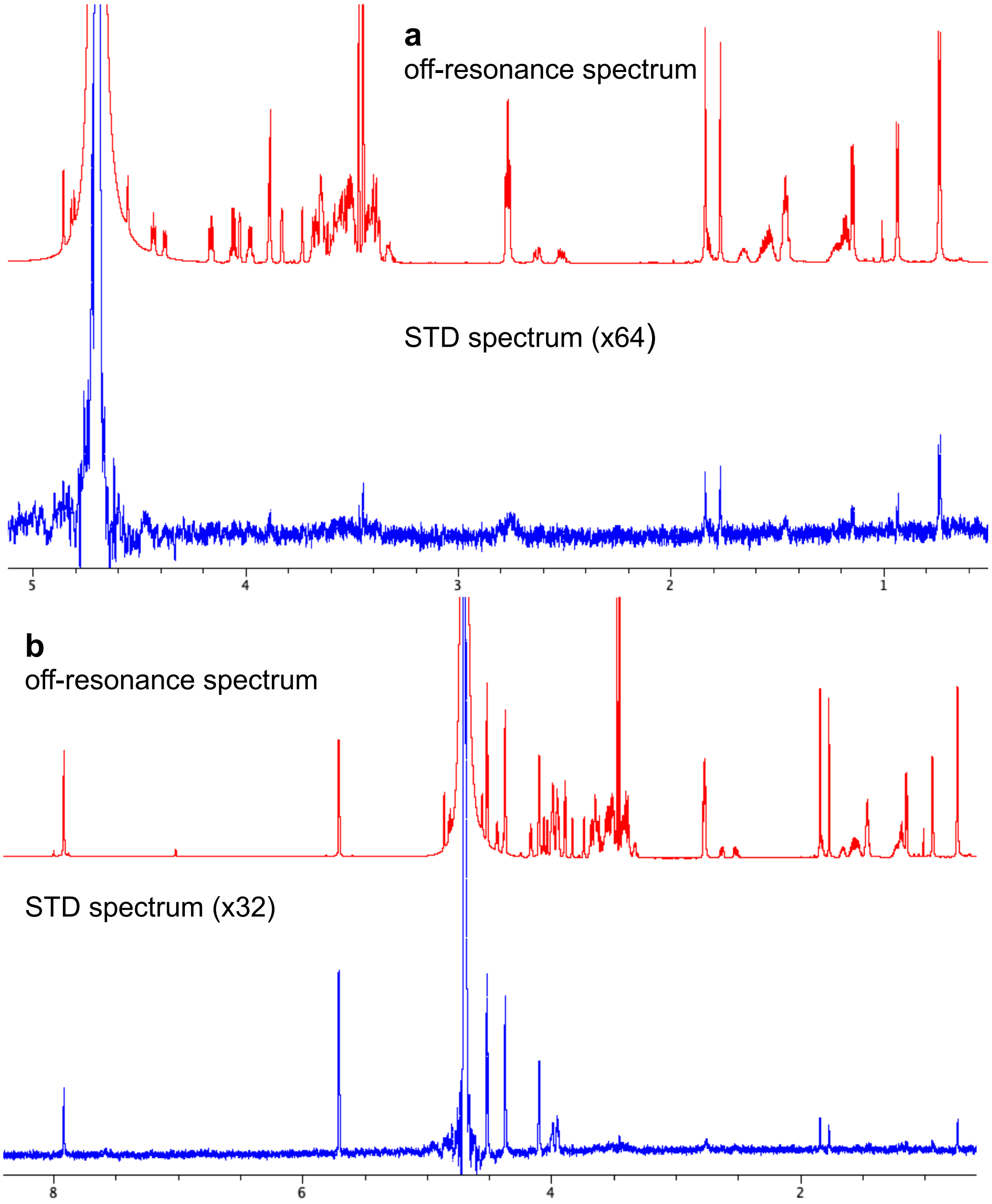

Figure S14. STD NMR analysis spectra for the M3N2-peptide in complex with FUT8 in the absence (a) and presence (b) of GDP. STD NMR spectra are scaled as indicated. Protein saturation was achieved by irradiation at -0.50 ppm.

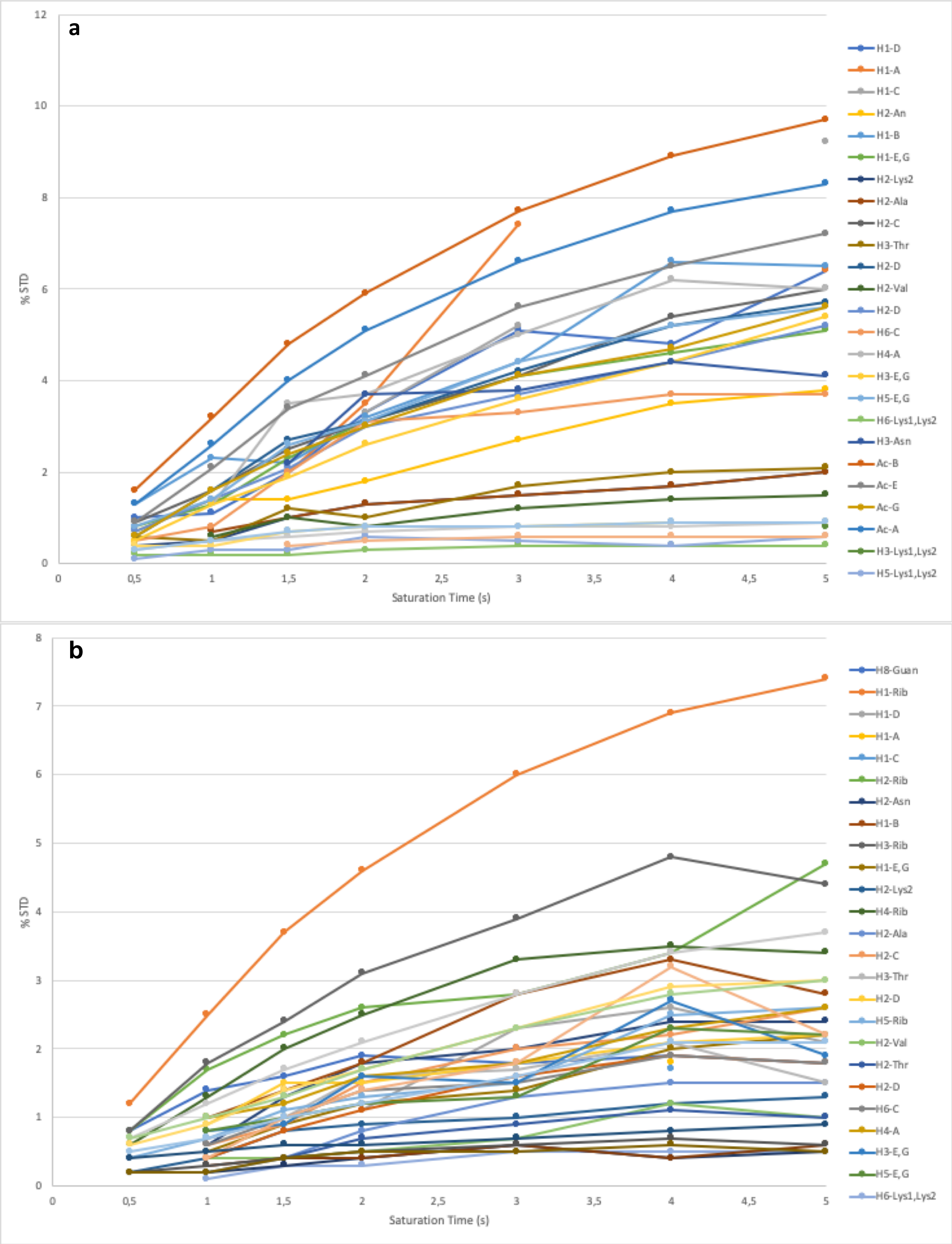

Figure S15. STD NMR build-up curves for the G0-peptide in complex with FUT8 in the absence (a) and in presence (b) of GDP.

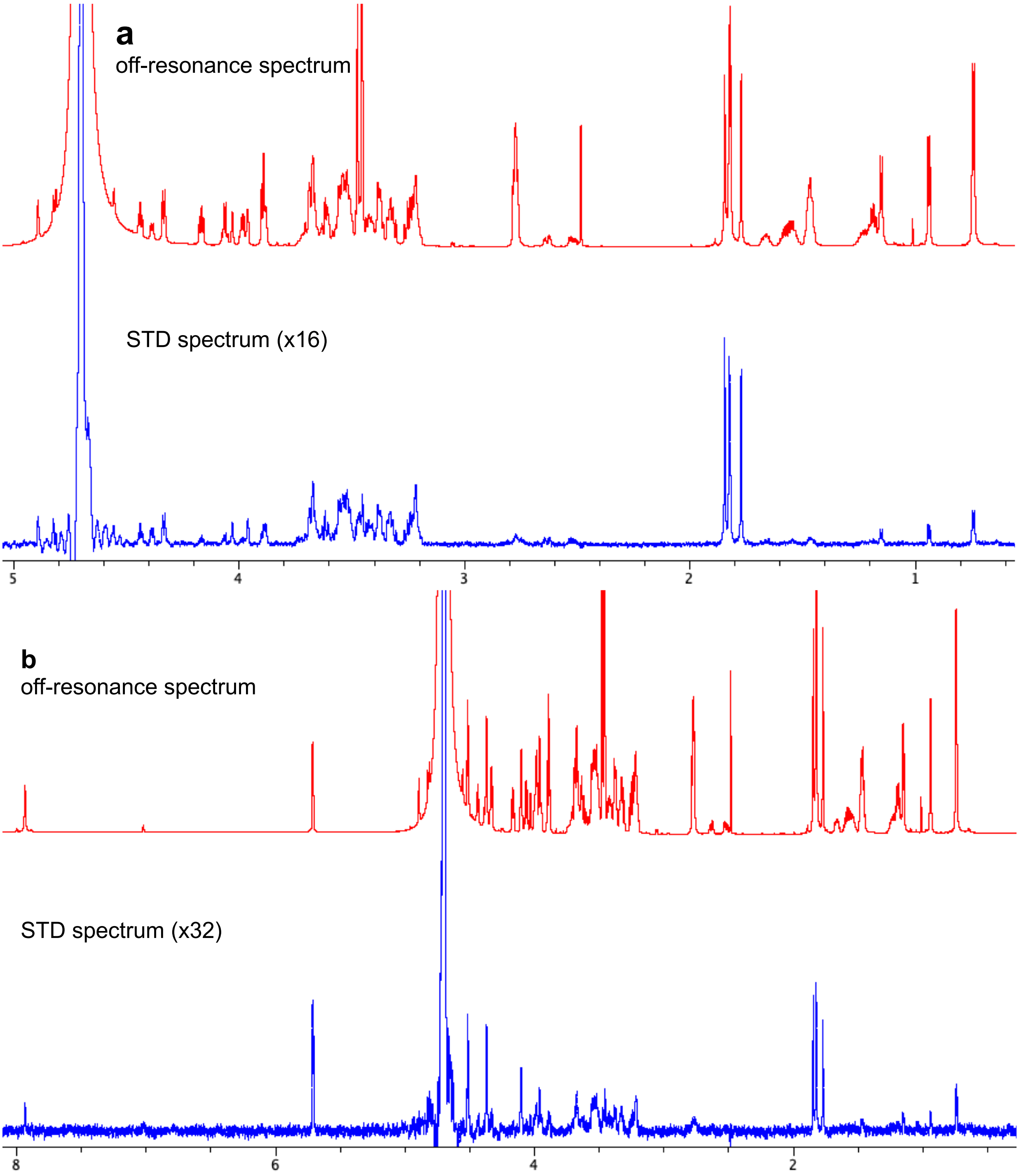

Figure S16. STD NMR analysis spectra for the G0-peptide in complex with FUT8 in the absence (a) and presence (b) of GDP. STD NMR spectra are scaled as indicated. Protein saturation was achieved by irradiation at -0.50 ppm.

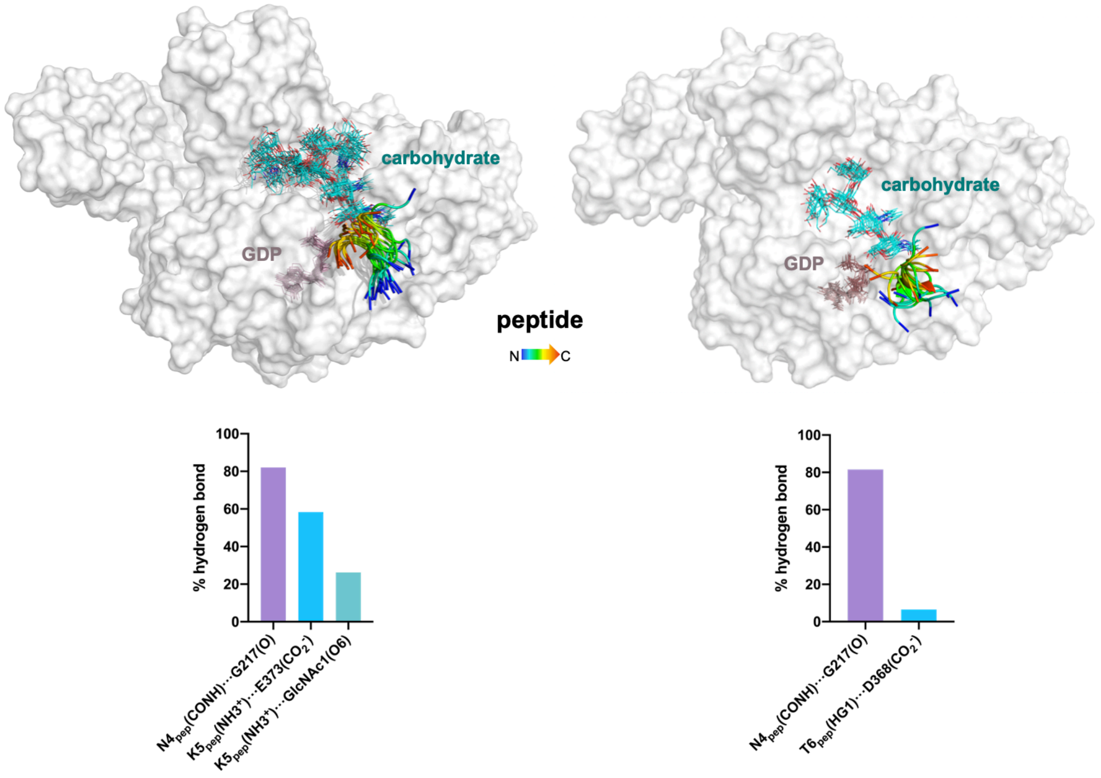

Figure S17. Structural ensembles derived from the 0.5 μs MD simulations performed on the FUT8 complexed to G0-peptide (left panel), and M3N2-peptide (right panel). In both cases, GDP was included in the simulations. Only the first frame of FUT8 is shown as a white surface. The glycan is shown as sticks and the peptide backbone as cartoon. The most populated hydrogen bonds between the peptide and FUT8 are also indicated.

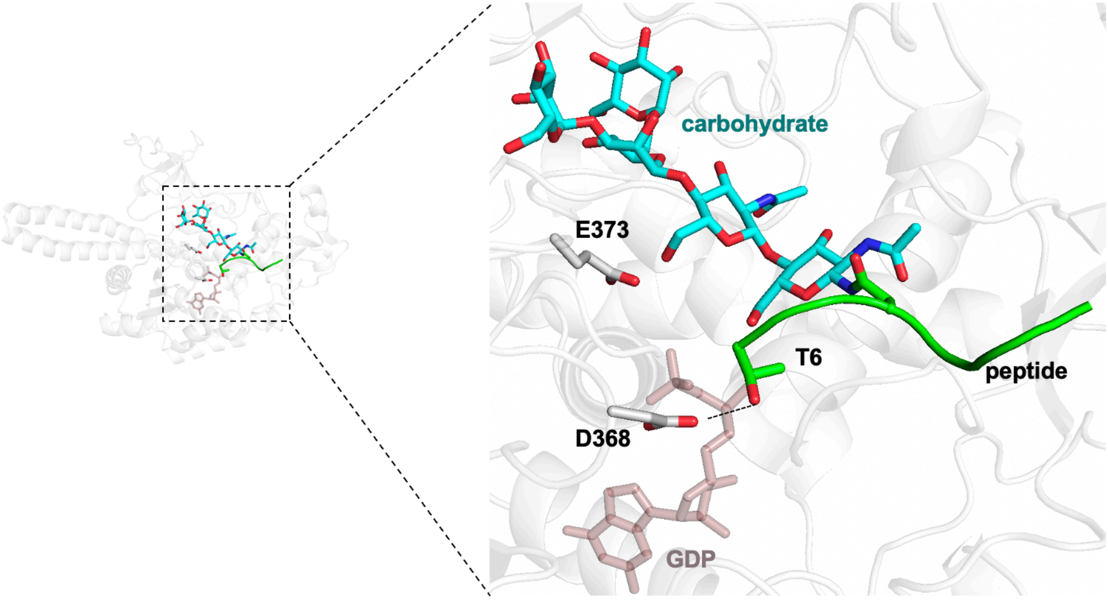

Figure S18. A representative frame obtained from the 0.5 μs MD simulations on the FUT8-GDP-M3N2-peptide complex, showing the hydrogen bond between the side chain of Thr6 and Asp368.

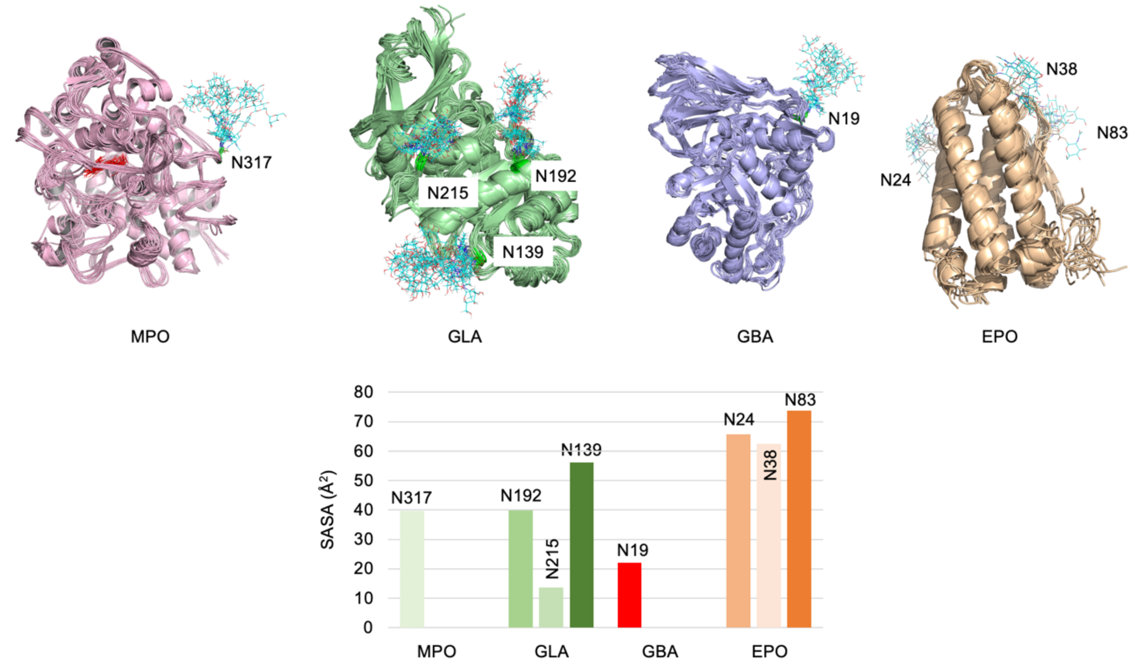

Figure S19. Structural ensembles obtained derived from 0.5 µs MD simulations of MPO (PDB entry 1D2V), GLA (PDB entry 1R46), GBA (PDB entry 5LVX) and EPO (PDB entry 1CN4). Proteins are shown in cartoon ribbons and the glycans as sticks. The solvent-accessible surface area (SASA) is shown for the hydroxymethyl group of the potentially fucosylated GlcNAc. The GlcNAc residue at Asn19, which exhibits one of the lowest SASA value is not fucosylated.
